# Cost of implementation and maintenance of maternal and perinatal death surveillance and response: a scoping review

**DOI:** 10.1186/s12884-025-08181-z

**Published:** 2025-10-06

**Authors:** Carly Malburg, Merlin Willcox, Tracey Sach, Mary Kinney, Patricia Akweongo, Animesh Biswas, Florina Serbanescu

**Affiliations:** 1 Division of Reproductive Health, National Center for Chronic Disease Prevention and Health Promotion, Centers for Disease Control and Prevention (CDC), 4770 Buford Highway, Atlanta, GA 30341 USA; 2 National Foundation for the Centers for Disease Control and Prevention, Inc, Atlanta, GA USA; 3 School of Primary Care, Population Sciences and Medical Education, Faculty of Medicine, University of Southampton, Southampton, UK; 4 Division of Global Surgery, Department of Surgery, University of Cape Town, Observatory, South Africa; 5 University of Ghana, Accra, Ghana; 6 United Nations Population Fund (UNFPA), Dhaka, Bangladesh

**Keywords:** Maternal perinatal death surveillance response, Economic evaluation, Costing analysis, Low- to middle-income counties

## Abstract

**Background:**

Globally, most countries have policies and guidelines requiring maternal and perinatal death surveillance and response (MPDSR), a system that can reduce avoidable maternal and perinatal deaths. Economic studies of MPDSR help inform resources to implement and sustain MPDSR at subnational and national levels. This review aims to scope the range of economic studies available and examine types of costs incurred by LMICs to implement and maintain MPDSR.

**Methods:**

We searched 11 electronic databases for key terms related to economics, maternal and/or perinatal death, health systems, surveillance, or audits/reviews. We included quantitative, qualitative, or mixed methods articles reporting costs of MPDSR, published in English, Spanish, or French during 2012–2023. Two independent authors screened titles and abstracts and extracted data. Costs were converted to the United States dollar price year 2024.

**Results:**

A total of 14,078 articles were systematically screened. Only 5 were included, as they reported costs of maternal and/or perinatal death surveillance and/or review. Of these only 3 reported itemized costs. None reported on costs of implementing recommendations. From the articles reporting itemized costs, in year 1 (start-up), the cost per death reviewed ranged from $113 to $5,758 and the cost per capita ranged from $0.40 to $1.11. In year 3, these declined to $86 to $577, and $0.26 to $0.66, respectively. The lowest cost per death was for conducting only maternal death reviews in health facilities. For community MPDSR, the lowest cost per capita was achieved by using a pre-existing functional household surveillance system to identify and investigate maternal and neonatal deaths. The highest cost was for establishing a new comprehensive death surveillance and review system, which investigated all deaths in women of reproductive age to identify maternal deaths only.

**Conclusion:**

Comparability was challenging because available literature was sparse and economic methods and study designs were heterogeneous. The cost–benefit of community death surveillance and review, compared to facility-based death notification and review, has not been clearly established. Better understanding of MPDSR costs is needed to prioritize and integrate MPDSR in health planning across system levels.

**Supplementary Information:**

The online version contains supplementary material available at 10.1186/s12884-025-08181-z.

## Key findings

1. What was known?
  Importance of this specific problem: Maternal and perinatal death surveillance and response (MPDSR) provides the data to inform strategies to prevent avoidable maternal and perinatal deaths. However, little is known about the economic costs and outcomes resulting from MPDSR implementation and maintenance at national and subnational levels. This may inform implementation and maintenance of MPDSR in low- and middle-income countries (LMICs) per the World Health Organization technical guidance.
  Key gap to address/aim of this paper: We searched for economic evidence on MPDSR. We reviewed the types of economic studies used to describe the implementation and maintenance of MPDSR systems in LMICs and report on the specific costs found.
2. What was done?
  High-level method: We conducted a scoping review to describe the types of costs and economic studies that are used to describe the cost associated with implementation and maintenance of MPDSR in LMICs. 
  Novel approach or analyses: No scoping review of implementation and maintenance costs of MPDSR has been previously conducted.
3. What was found specific to strengthening mpdsr implementation & action?
  Key result findings: Only five of 14,078 papers from 11 databases met our search criteria, and only three contained full implementation and maintenance cost breakdowns. No full economic evaluation of MPDSR in LMICs was reported in the literature. We documented variation in costs per death reviewed and per capita in different programs. In three studies combining surveillance of maternal, perinatal, and neonatal deaths we observed reduced cost per death reviewed, compared to surveillance of maternal deaths alone. While community death surveillance and review are more comprehensive than facility-based surveillance alone—because the former the potential to include all deaths—there was one study that demonstrated whether community- or facility-based MPDSR or their respective costs have a greater impact on implementation and maintenance of the system.
4. What are the implications for strengthening MPSDR implementation & action?
  Action in programs and/or measurement now: Developing approaches for reporting costs specific to MPDSR, building skills, and adhering to standardized analyses can demonstrate the economic value of sustainable MPDSR systems. Capacity-building is occurring to compare the start-up and maintenance processes across health system levels, populations, and countries to further enhance MPDSR systems in LMICs.
  Future research gaps: Comparing implementation and maintenance costs of different MPDSR systems is currently challenging because of varying coverage, approaches, and needs of each system. Identifying critical costs and cost-saving approaches could increase the quality and effectiveness of MPDSR. To inform MPDSR in resource-limited settings, data on cost-effectiveness of different approaches can help make evidence-informed decisions.

## Background

Reducing maternal and perinatal mortality in low- and middle-income countries (LMICs) is a global public health priority [1]. This is recognized by the Sustainable Development Goals (SDGs) that aim to reduce the maternal mortality ratio globally to less than 70 per 100,000 live births by 2030 [2]. The SDGs similarly aim to reduce the neonatal mortality rate to under 12 per 1,000 live births and the under-5 mortality rate to under 25 per 1,000 live births [3] The World Health Organization (WHO) published global technical guidelines on conducting maternal death reviews in 2004, later expanding their scope to maternal death surveillance and response (MDSR) in 2013 [4, 5]. The goal of the 2013 guidance was to introduce the critical concepts of MDSR, including specific instructions for implementing each surveillance component to prevent future avoidable maternal deaths [5]. Guidelines for perinatal death audits were released by WHO in 2016, and a joint operational guidance and tools for maternal and perinatal death surveillance and response (MPDSR) implementation was published in November of 2021 [6, 7].

The term 'MPDSR' was formally adopted by WHO in recent years [7]. Previously, guidance documents on maternal and perinatal death audits were distinct [5, 6]. MPDSR is a complex intervention compounded by the wide variety of implementation strategies across countries [8–10]. Table 1 provides a simplified overview of mortality definitions and describes what MPDSR is, how it works, and the ways it has been implemented in health systems.

**Table 1:** What is MPDSR?

Definitions: Maternal death – death of a woman from any cause related to or made worse by pregnancy or its management. This includes deaths during pregnancy, during childbirth, or within 42 days of pregnancy ending, regardless of the pregnancy's length or location. Accidental or incidental causes are not included [11] Perinatal death – a stillbirth of weight > 1,000 g after at least 28 weeks gestation [11] Neonatal death – deaths among live births during the first 28 completed days of life which can be further sub-divided into early neonatal deaths (deaths between 0 and 7 completed days of birth) and late neonatal deaths (deaths after 7 days to 28 completed days of birth) [11]	
Aim of MPDSR: MPDSR is a systematic process within healthcare systems. It involves identifying, notifying, and reporting maternal and perinatal deaths ("surveillance”). Information is gathered about these deaths, and this is reviewed to identify avoidable factors and make recommendations to avoid similar issues in the future. Implementation of these actions to enhance the quality of care and service delivery is the “response” [8]. The aim is to prevent future avoidable maternal and perinatal deaths	Implementation as an intervention: Implementation of MPDSR as a quality enhancement strategy is intricate, adaptable, and contingent on contextual factors. It operates across different tiers of the healthcare system—national, subnational, and facility levels—and is influenced by factors at micro, mezzo, and macro levels. Optimal implementation reduces maternal and perinatal mortality [8]. However, systems may be ineffective or may even be harmful [8, 9, 12]
MPDSR components: MPDSR serves as an overarching concept, encompassing several distinct yet interconnected components. Different settings or programs may implement one or more of these, as LMICs are at different stages of implementing MPDSR (national or subnational, including all health facilities or only selected facilities, including communities inconsistently) [1, 4, 6, 9, 10, 13, 14]. Components include: - Notification of deaths in health facilities - Review meetings in health facilities - Review meetings at district and/or national level - Surveillance system for notification of deaths at community level - Verbal autopsy (questionnaires/interviews to ascertain cause of death in the community) - Social autopsy (questionnaires/interviews to identify social factors at the family and community level that could have averted the death) - Community death review meetings - Confidential enquiry (a systematic process of multi-disciplinary, anonymous review of all or a sample of defined cases occurring in a defined geographical area during a defined period) - Implementing recommendations for quality improvement [15] - Monitoring and evaluation for system strengthening The various descriptions of this intervention underscore its nuanced and intricate nature [7, 14, 16]; and yet different contexts require different approaches and components [7]	

In 2018, 126 countries of the 150 surveyed by WHO reported that they have a policy or guidance for MPDSR [13]. When properly implemented, MPDSR can increase access, improve quality of care, and reduce institutional maternal and perinatal mortality [8, 10, 14, 17]. Insufficient resources or poor resource management are common barriers for failure to implement or maintain MPDSR systems in LMICs [8–10]. Very few studies assessing MPDSR quantified the actual costs of surveillance implementation and maintenance within a health system [8]. Even fewer studies reported the costs of implementing and following up on the recommendations stemming from an MPDSR review [18].

We conducted a scoping review with the aim of documenting the available literature on only the costs and cost-effectiveness of implementing and maintaining MPDSR within a health system.

## Methods

### Study design

We followed Arksey and O’Malley’s 6-stage methodological framework as the main guideline [19]. Our strategy was developed by studying published scoping reviews centered around maternal and perinatal death reviews, maternal health topics and economic evaluations [9, 14]. To refine the search we used Medical Subject Headings (MeSH) terms and free-text key words such as “maternal health,” “stillbirth,” “neonatal health,” “program assessment,” “surveillance,” “maternal death review,” “perinatal death review,” “economic evaluation,” “costing analysis,” and “low-income and middle-income countries,” including the name of individual countries. The full strategy is available in Supplementary Table 1.

Our search adhered to the Preferred Reporting Items for Systematic Reviews and Meta-Analyses (PRISMA) guidelines [20]. We included peer-reviewed articles and grey literature published from January 2012 through January 2024. The search period was selected based on the publication of the 2013 MDSR WHO guidelines, prior to which few LMICs had policies in place to support MDSR. LMIC countries were defined according to the World Bank income groupings in 2012 [21]. Studies were identified through searching the following electronic databases: Medline (OVID), Embase (OVID), Global Health (OVID), Cochrane Library, CINAHL (Ebsco), Scopus, Sociological Abstracts on ProQuest, EconLit, Global Index Medicus, ProQuest Dissertations & Theses Global, and OpenGrey. We searched for literature in English, French, or Spanish published in 2012–2023.

### Study selection – peer-reviewed articles

We targeted all peer-reviewed literature that focused on facility- or community-based MDSR or MPDSR systems. We included all quantitative, qualitative, or mixed methods studies assessing costs of system implementation and/or maintenance in LMICs.

### Study selection – grey literature

We performed an expanded search targeting reports, theses, project documents, and web publications. This additional search intended to gather program costs related to facility or community-based implementation of MDSR or MPDSR systems in LMICs that were not published in peer-reviewed journals. Reports or published materials that did not include costs were excluded from the search.

### Exclusion criteria

We excluded studies involving animals, those set outside of LMICs, editorials, commentaries, viewpoints or essays, protocol- or abstract-only articles, review articles, articles not related to maternal and perinatal health interventions/programs, and all articles published prior to 2012 or in a language other than English, French, or Spanish.

### Article screening

We exported all articles into the EndNote version 20 (Clarivate, Philadelphia, PA, USA) reference management software and uploaded them into Covidence (Covidence, Melbourne, Australia), a systematic review management software [22, 23]. We used EndNote to collate articles from the various databases and used Covidence for deduplication, article screening, and assessment for eligibility. Titles and abstracts were screened against inclusion and exclusion criteria by two researchers independently (CM and FS). If a study was not excluded by both reviewers at the abstract screening stage, we conducted a full-text review. The full-text review and eligibility decisions were made independently by two researchers (CM and FS). Discrepancies were discussed and adjudicated by a third researcher (MW).

### Data extraction and analysis

Data extraction was completed independently by four reviewers (CM, TS, MW, PA) using standardized forms in Covidence. Differences were resolved by one independent consensus reviewer (FS). We contacted the authors of all included articles to both clarify their methods and request additional cost information and data from their analyses. Two authors responded and provided additional method clarification and cost related data [24, 25].

The following elements were extracted from each article: study author(s), country of implementation, study aim and population, surveillance funding sources, method for identifying deaths, number of deaths reported and reviewed, MDSR or MPDSR system components evaluated, study type, economic analysis type, implementation cost(s) per death reviewed, cost categories, and the economic perspective of the study.

We used a narrative analysis to describe the scope, design, MDSR or MPDSR and economic components, and main findings for the literature included in this review. We also extracted costs in the local currency and price year so that we could adjust: costs to a common price year, and currency to aid comparison across studies that we included. We used the CCEMG-EPPI-Centre Cost Converter v.1.4, a web-based tool utilizing gross domestic product deflator indices and purchasing power parities conversion rates, which automates cost adjustment to target currency and price year [26]. As such, in this paper, all costs are presented in United States dollars (USD) for the 2024 price year. Original costs extracted from the papers can be found in Supplementary Table 2.

### Assessment of reporting completeness

The type of economic study conducted was assessed according to an adapted classification (Table 2) [27].

**Table 2:** Description of full and partial economic evaluations

Full economic evaluations: • Studies that compare two or more alternatives* in terms of both costs (inputs) and consequences (outputs) • All economic evaluations measure costs in monetary units • The type of economic evaluation is determined by the choice of outcome		
Types of full economic evaluation: • Cost–benefit analysis • Cost-utility analysis • Cost-effectiveness analysis		Measurement of consequences, i.e. outcomes measured in: • Monetary units • Health years. (e.g. quality-adjusted life-years) • Natural units. (e.g. life-years saved, point reduction in blood pressure, etc.)
Partial evaluations: • Studies that consider just costs or just outcomes or both but do not compare to another alternative Or • Studies that do compare two or more alternatives but only in terms of costs or consequences, not both		
Types of partial evaluation: • Outcome description • Cost description • Cost-outcome description • Effectiveness evaluation • Cost analysis	Study focus: • Examining only outcomes for a single alternative • Examining only costs for a single alternative encompassing micro costing (bottom up) or macro costing (top down) study designs • Examining both costs and outcomes for a single alternative • Comparing only effectiveness for two or more alternatives • Comparing only costs for two or more alternatives	

^*^Note: where an alternative is an intervention, treatment, service, or policy. Adapted from Drummond et al. 2015 [27]

We also attempted to evaluate the studies according to the Consolidated Health Economic Evaluation Reporting Standards (CHEERS), a 28-item checklist used to assess the reporting quality of health economic evaluations [28]. CHEERS is primarily intended for full economic evaluations, but assessing a number of its items is also relevant for partial evaluations such as cost analyses. To interpret economic evidence appropriately, it is important to critically appraise the perspective and methods reported, in addition to considering the transferability of the evidence to settings other than the one in which it was conducted [28]. We used the CHEERS reporting standards to: inform the creation of our data extraction form in Covidence, and guide our analysis of MPDSR costs.

## Results

### Screening

**Fig. 1:**
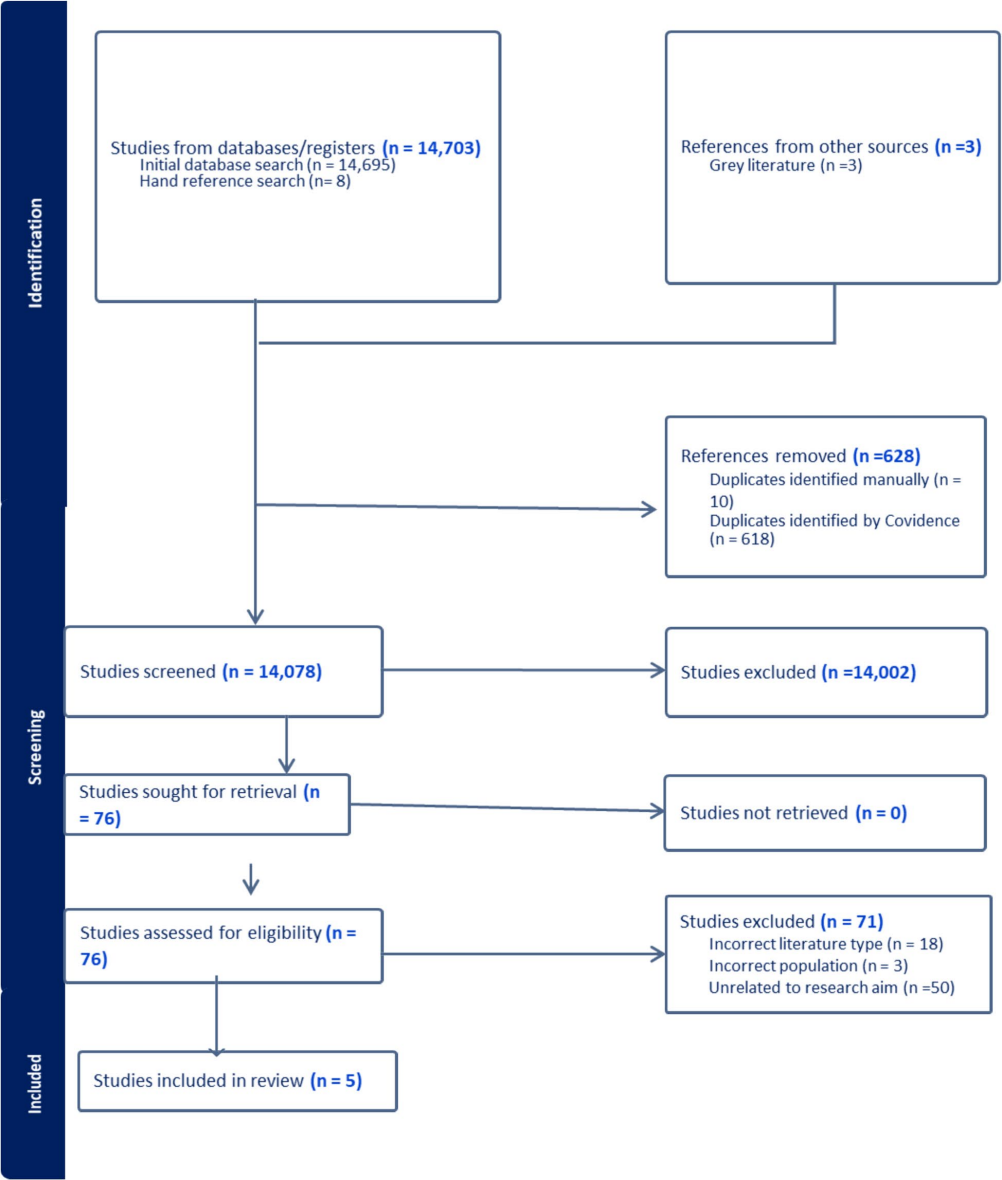
Preferred Reporting Items for Systematic reviews and Meta-Analyses (PRISMA) flow diagram. Note: PRISMA diagram was generated by covidence software

Our search strategy yielded 14,703 articles. After removing duplicates (*n* = 628), 14,078 articles were screened (Fig. 1). During title and abstract screening, 14,002 articles were excluded for not meeting the inclusion or exclusion criteria. In full text screening, 76 articles were screened. However, 71 articles were excluded, primarily because the article was unrelated to research aims (*n* = 50), was an incorrect literature type (*n* = 18), or included an excluded population (*n* = 3). Our final dataset consisted of five papers, three from the database search and two from our grey literature search.

### Characteristics of included studies

Table 3 presents the characteristics of the five papers included in the final analysis. The MDSR or MPDSR systems included in this analysis were in Zimbabwe (*n* = 2), Bangladesh (*n* = 1), India (*n* = 1), and Uganda (*n* = 1). Two articles reported costs of facility-level reviews of maternal deaths only [29, 30], one reported costs of only verbal autopsies (VAs) of all child and adult deaths at the community level [31], and two reported costs of a comprehensive system of community surveillance; community verbal and social autopsy; and facility-based reviews of maternal, perinatal, and neonatal deaths [24, 32]. Two studies received support and funding from government entities [24, 29, 30], whereas three were mainly donor-funded programs [25, 31]. No papers (1) included unanticipated costs of implementing and maintaining a MDSR or MPDSR system or (2) performed comparative cost analysis or full economic evaluation of MDSR or MPDSR. All studies reported costs at a sub-regional level; three studies included only one district [24, 29, 30], while two studies examined costs in two or more districts [25, 31].

**Table 3:** Characteristics of studies included in the analysis, *n* = 5

Reference	Country	Study settings	Year of data collection	Level of health system	Type of death reviewed	Implementing agency	Implementation funding source	MPDSR components examined	Study type^a^	Economic analysis design^b^	Economic perspective^c^
Biswas et al. (2016) [24]	Bangladesh	Thakurgaon district	2010–2012	Community and facility	Maternal and neonatal	Bangladesh government (DGHS and DGFP), CIPRB and UNICEF	External (multinational, Canadian CIDA and DFID)	Identification and reporting of deaths; death reviews; response	Quantitative	Micro costing (bottom up)	Program
Joshi et al. (2015) [31]	India	45 rural villages Godavari district of the Andhra Pradesh region	2003–2007	Community	Adult and child	The Byrraju Foundation	External (non-government organization)	Identification and reporting of deaths; death reviews; verbal autopsy	Quantitative	Micro costing (bottom up)	Program
Serbanescu et al. (2016) [25]	Uganda	Kibaale, Kabarole, Kyenjojo, and Kamwenge districts	2013–2014	Community	Years 1, 2: Maternal Year 3: Maternal and neonatal	Uganda Ministry of Health, Baylor Uganda, USAID, CDC	Public–private partnership	Identification and reporting of deaths; verbal social autopsy	Quantitative	Micro costing (bottom up)	Program
Mutsigiri-Murewanhema et al. (2017) [30]	Zimbabwe	Mutare district	2014	Facility	Maternal	Zimbabwe government	Internal (government)	Identification and reporting of deaths; death reviews; response	Mixed methods	Macro costing (top down)	Program; level of health facility; government
Tapesana et al. (2017) [29]	Zimbabwe	Sanyati district	2015–2016	Facility	Maternal	Zimbabwe government	Internal (government)	Not stated; evaluation focused on system attributes	Mixed methods	Macro costing (top down)	Program

*Abbreviations*: *CDC* Centers for Disease Control and Prevention, *CIDA* Canadian International Development Agency, *CIPRB* Center for Injury Prevention and Research, Bangladesh, *DFID* Department for International Development, *DGFP* Directorate General of Family Planning, *DGHS* Directorate General of Health Services, *USAID* United States Agency for International Development

^a^Study type includes the methodology used to collect data about costs (i.e. quantitative, qualitative, mixed methods)

^b^Economic analysis design refers to the methodology the article used to analyze data on reported costs

^c^Economic perspective refers to the frame of the costs reported in the article, such as costs related to organization, staff, program, society, etc.

### Quality assessment

All studies were partial economic evaluations. No studies included enough items from the CHEERS checklist for us to apply it. For example, to compare costs per death reviewed, we had to obtain additional information from the authors [24, 25]. This additional information included more details about cost categories and the number of deaths (maternal and perinatal) reviewed per year. Consistent cost reporting in three articles included costs related to training, infrastructure and capacity building, project management, and costs per death reviewed. Only two articles reported the cost per maternal/perinatal death averted [24, 25].

### Reported costs

The two studies in Zimbabwe assessed the attributes of the entire MDSR system at the district level and reported the cost per maternal death identified through MDSR [29, 30]; neither article explicitly stated whether the reported costs included only death notification or also included the death review. The three other papers reported itemized implementation and maintenance costs for MPDSR systems by cost categories over 3 [24, 25, 31] or 4 years [26]. Table 4 describes the total reported costs, cost per death reviewed, and cost per capita for all five included studies. Supplementary Tables 2 and 3a–c describe these same costs broken down by cost category. Among Supplementary tables, Table 2 describes the total and itemized costs in the currency originally reported, Table 3a details the costs in year 1 (start-up year), and Tables 3b and 3c detail the costs in years 2 and 3 (maintenance costs). Not included in the supplementary tables are costs from the Zimbabwean studies because studies reported only an average cost per maternal death review [29, 30], with no itemized costs, and no specification regarding start-up or maintenance costs.

**Table 4:** Reported total costs per death reviewed and per capita in USD 2024 for included articles, *n* = 5

Author	Country	Population^a^	Deaths reviewed^a^	Total cost^b^	Cost per capita^b^	Cost per death reviewed^b^
Tapesana^c^ [29]	Zimbabwe	215,842	25	NA	NA	$ 314.00
Mutsigiri-Murewanhema^c^ [30]	Zimbabwe	NA	32	NA	NA	$ 37.00
Joshi, year 1 [31]	India	185,628	1,496	$ 168,547.62	$ 0.91	$ 112.67
Joshi, year 2 [31]	India	185,628	1,430	$ 122,334.22	$ 0.66	$ 85.55
Joshi, year 3 [31]	India	185,628	1,430	$ 122,334.22	$ 0.66	$ 85.55
Biswas, year 1 [24]	Bangladesh	1,400,000	1,590	$ 564,984.27	$ 0.40	$ 355.34
Biswas, year 2 [24]	Bangladesh	1,400,000	1,667	$ 446,774.49	$ 0.32	$ 268.01
Biswas, year 3 [24]	Bangladesh	1,400,000	1,382	$ 358,288.56	$ 0.26	$ 259.25
Serbanescu, year 1 [25]	Uganda	1,278,004	247	$ 1,422,113.00	$ 1.11	$ 5,757.54
Serbanescu, year 2 [25]	Uganda	1,278,004	177	$ 709,218.29	$ 0.55	$ 4,006.88
Serbanescu, year 3 [25]	Uganda	1,278,004	1,302	$ 751,784.24	$ 0.59	$ 577.41

The authors focused on assessing the entire MDSR system at the district level and we were not certain what components were costed in the average costs per death reported by the system. The authors of those papers also did not report if these average costs were for implementation or maintenance of the system. We reached out to Tapesana and Mutsigiri-Murewanhema et al. for clarification but did not hear back from them

*Abbreviations*: *USD* United States Dollar, *NA* Data not available, *MDSR* Maternal death surveillance and response

^a^Population and number of deaths reviewed are taken directly from what was reported by the respective authors. Population available only for year 1 and assumed constant for years 2 and 3, where applicable

^b^All costs are converted from how they were originally reported to $USD 2024 and adjusted for purchasing power parity and gross domestic product using the CCEMG-EPPI-Centre Cost Converter v.1.4 (ioe.ac.uk) tool

^c^We assumed the reported costs by Tapesana et al. and Mutsigiri-Murewanhema et al. are per death notified and reviewed

### Cost categories

Three articles reported costs in most of the following categories of MPDSR activities: training, tool development, meetings, infrastructure/capacity, project management, data collection (community and facility), and monitoring [24, 25, 31]. Table 5 outlines what costs from each article are included in each category. These costs are taken directly from the respective articles [24, 25, 31].

**Table 5:** Reported categories and associated costs by select articles, *n* = 3

Cost category	Cost category description		
	Joshi et al. [31]	Biswas et al. [24]	Serbanescu et al. [25]
Training	Costs associated with developing protocols and tools, conducting workshops, and supporting participants’ attendance at training sessions	Costs associated with conducting workshops and supporting participants’ attendance at training sessions	Training of VHTs, parish coordinators, and health extension personnel to notify deaths of WRA, screen them, and conduct VASA. Physician trainings to review VASA and certify and code the cause of death. Includes baseline and year 2 and 3 refresher trainings
Tool development	Included in training costs	Costs associated with development of surveillance tools and training manuals	Included in project management
Meetings	Not reported	Costs of conducting periodic staff meetings and conducting review meetings at the sub-district (upazila) and district levels	Transport costs for VHT, health extension workers, and parish coordinators to attend parish meetings (monthly in year 1 and quarterly in years 2 and 3)
Infrastructure, capacity building	Computers, printers, phones, furniture, and local travel expenses. Office space provided in-kind by the study partner (Byrraju Foundation)	Costs incurred for office setup (equipment and supplies), office monthly expenses, and communications	VHTs’ supplies (bicycles, equipment for field work, mobile phones). Office costs provided in-kind by the implementing partner (Baylor Uganda)
Project management	Salaries for the project coordinator and field coordinator, costs for photocopying VAs, and courier costs. Data analyses and research associated costs not included, as the paper aimed to describe non-research components	Salaries for the principal investigator, project management staff, district coordinator, and data entry supervisor	Salaries for project coordinators, district coordinators, and data entry supervisor. Printing of tools, office supplies, and communication expenses for the district coordination teams
Community data collection	Salaries for non-physician healthcare workers and the physicians' fee for certification and coding of VAs	Costs associated with community and facility notification of maternal and neonatal deaths; cost associated with travel and per-diem for conducting VAs and VASAs for community deaths; cost associated for conducting death reviews in health facilities	Costs of VHTs’ monthly household visits (travel costs and per diem); VHTs’ transportation costs to parish meetings; extension health workers’ costs for conducting pregnancy screenings and completing VASA with the families of deceased WRA; physicians' fees for VASA certification and coding
Monitoring	Not reported	Salary support for M&E personnel, including: travel costs for conducting notification quality checks; completion of VAs and VASAs; data entry; and data file management	Salary support for M&E officers (1–2 in each district); travel costs for conducting notification quality checks; completion of VASAs; data entry; and data file management

Joshi et al. [31], Biswas et al. [24], and Serbanescu et al. [25] were the only papers to report cost categories and itemized costs. Cost categories as described by authors, and with follow-up from Biswas and Serbanescu, for additional description of itemized costs. Biswas et al. were the only authors to collect facility-level itemized costs

*Abbreviations*: *M&E* Monitoring and evaluation, *VHTs* Village health teams, *VA* Verbal autopsy, *VASA* Verbal and social autopsy, *WRA* Women of reproductive age (12–49 years of age)

### Costs of facility-based maternal death reporting and review

The two Zimbabwean studies evaluated the attributes of the maternal death surveillance system at the district level over 1–2 years [29, 30]. Although the surveillance system evaluated included facility- and community-based death notification and reporting and community VAs, the authors did not include the community costs. Mutsigiri-Murewanhema et al. evaluated the performance of the maternal mortality surveillance system in the Mutare district in 2014 [30]. They estimated that each maternal death review costs approximately $37 (USD 2024) but did not explain their methods or what was included in this cost [30]. Tapesana et al. evaluated the MDSR system in Sanyati district in 2015–2016 [29]. They estimated the total cost of reviewing an institutional maternal death was about $314 USD 2024 per death [29]. They accounted for the time it took for a nurse to complete the data collection and death notification, write the maternal death report form, report the death, deliver the death notification form at the district level, and attend the death review meetings at the district hospital [29].

### Costs of community-based verbal autopsies and MPDSR

Joshi et al. provided a comprehensive description of costs for: (1) community-based reporting and VA interviews (conducted by “non-physician health workers”) for all child and adult deaths, and (2) assignment of cause of death by two independent physicians [31]. The authors reported costs including infrastructure, training, salaries of the management team, and filed work costs for data collectors and physicians who reviewed the VAs and assigned causes of death [31]. The mortality surveillance identified 5,895 adult and child deaths over a 4-year period from October 2003 to September 2007, of which 96.7% (5,786) had a VA completed [31]. These efforts enabled economies of scale, resulting in a low cost per death ($113 in year 1, $86 in years 2 and 3) [31]. However, the cost per capita ($0.91 in year 1, $0.66 in years 2 and 3) was higher compared to that in the other studies, because all deaths were reviewed, not only maternal and perinatal deaths [31]. There were no death review meetings either in communities or health facilities, there was no component of making or implementing recommendations, and data analyses costs were intentionally excluded [31]. Maternal and neonatal deaths (which would represent only a small fraction of the deaths with VAs) were not studied separately, so the authors did not conduct a standard MPDSR system evaluation.

Only two studies reported costs of community and facility-based maternal and neonatal death reviews [24, 25]. In Bangladesh, the authors described the implementation and maintenance costs of the maternal and neonatal death review (MNDR) in one district over a period of 3 years (2010–2012). The system was implemented with donor support and build on existing district personnel and routine community surveillance functions. The system included community notification, VA, community death review meetings (called “social autopsy”), and facility notification and reviews [24]. Community health workers (CHWs) completed death reviews and surveillance as part of their designated roles, which reduced system costs and resulted in program cost savings. The paper provided itemized costs per activity and per death reviewed but did not report how they calculated these costs. Follow-up information on reported itemized costs and cost calculations were obtained from the first author.

In Uganda, Serbanescu et al. reported the MDSR lessons learned from the Saving Mothers Giving Life (SMGL) initiative that was implemented in four districts in 2013 [25]. While there was no formal economic analysis of the surveillance system implemented and maintained by SMGL, the authors provided the unpublished detailed budget. The goal of the initiative was to improve maternal and neonatal survival rates in the four districts by implementing different evidence-based practices to work alongside practices that were a part of the existing health systems. At the initiative’s onset in July 2012, a retrospective Reproductive-Age Mortality Study (RAMOS) was conducted to capture community-level maternal deaths. Trained village health teams (VHTs) used community registers to identify and compile lists of deaths among women of reproductive age (WRA) in their communities. Households with WRA deaths were visited by a trained VA team. If the death occurred during pregnancy or delivery, or within 2 months of delivery, the team collected information about the circumstances of death and contributing factors, using a comprehensive verbal and social autopsy (VASA) tool [32]. Starting with 2013, the districts set up a prospective MDSR system modelled on RAMOS, where VHTs identified and reported WRA deaths to sub-district health coordinators monthly. These initial costs were very high ($5,757 per death reviewed) because of extensive capacity building and training in year 1, and because this comprehensive system meant that 5–6 WRA deaths were investigated for each confirmed maternal death. Beginning in 2015, Uganda VHTs supported the integration of neonatal death surveillance into the MDSR system at no additional cost, so the cost per death reviewed reduced by a factor of 10 (to $577). The total cost per capita per year was $1.11 in year 1 but halved in years 2 and 3.

## Discussion

### Summary of findings

This review highlights a gap in the literature regarding costs to implement and sustain MPDSR systems. Out of 14,703 articles, we identified five studies with partial evaluations of MPDSR costs from four LMICs [24, 25, 29–31]. Two costed a full MPDSR system, including both community- and facility-based notification and reviews for maternal and neonatal deaths, but none counted the cost of implementing recommendations [24, 25]. One study reported stillbirths, though not purposefully included in the surveillance [31]. This reflects that most MPDSR systems prioritize maternal and neonatal outcomes and do not include stillbirths [33]. We found no studies that assessed the cost of MPDSR implementation at a national level; yet 126 countries report on having MPDSR systems in place [13].

Three of the studies included in this review were fully or partially funded by external donors and supported by government ownership [24, 25, 31]. Reporting of the funding source for MPDSR implementation—and whether there is government ownership of the MPDSR system—are inconsistently reported in the broader literature [8, 34]. When it is reported, projects strictly funded by external funds and actors (e.g. United Nations agencies, donors, etc.) are likely to experience a strong influence of the donors in MPDSR implementation, particularly due to the large investments needed to support implementation [8]. However, these projects, specifically those with little to no government ownership or buy-in, are rarely sustained [34].

In-depth knowledge about each MPDSR system is key when interpreting reported cost data in published articles. In Bangladesh, MPDSR was integrated into an existing functional government system which included regular household visits from CHWs [24]. In Uganda, the costs per death reviewed were over tenfold higher in the inception phase of SMGL, because a functional system of household surveillance needed to be created [25]. Initially the surveillance focused on comprehensive identification of all maternal deaths, by investigating deaths of all WRA, about 80–85% of which were not maternal deaths. Without this comprehensive system, about 35% of maternal deaths would not have been identified [25]. Further, the SMGL intervention was associated with a 43% reduction in maternal mortality [32]. The cost per death averted in Uganda was reported in a separate SMGL economic analysis using the itemized data published earlier [32]. Johns et al. found the cost of death averted through SMGL-supported interventions, including rigorous monitoring, evaluation, and surveillance, was $13,154 (in USD 2024), with an incremental cost of $226 per life year gained [32]. After perinatal deaths were added, costs per death reviewed declined but were still double the cost in Bangladesh. This was probably because of the exhaustive system of investigating all WRA deaths, and because the extensive CHW infrastructure in Uganda—about 3,800 VHTs visited 100–300 households each and reported monthly on the number of deaths among WRA identified in the previous 30 days—was not government funded. The Bangladesh system was integrated into the government health system and employed government health workers in their existing roles, whereas SMGL set up a novel surveillance system.

None of these studies present a full economic evaluation for MPDSR and related processes, as a full economic evaluation requires comparing the inputs and outputs of two or more alternative interventions [27]. Only one study provided the detailed costs in each category of the MPDSR cycle [31]. Performing full economic evaluations and knowing the full details of the reported costs and outcomes would allow for true comparability of value for cost across systems. These data may subsequently inform effective implementation and maintenance of the MPDSR system.

There are many potential reasons why we found few studies to evaluate. For instance, there are perceptions that MPDSR requires no additional costs; instead the costs are embedded within existing health systems or cost related decisions are made at a governmental level [18, 35]. Recent reviews on cost and cost-effectiveness of quality improvement collaboratives [36] as well as audit and feedback interventions [37] also find similar results—including a scarcity of studies and inconsistent reporting [38]. For the quality improvement collaborative review, only eight studies were identified, five of which included economic evaluations suggesting related interventions were cost-effective [36]. The systematic review of audit and feedback interventions found 35 studies mostly from developed countries, of which 26 were perceived as potentially cost-favorable, indicating the benefits of the intervention justify the costs, including 7 that were cost-favorable [37] – and this may reflect publication bias. As with our study, these reviews found variations in methods and approaches, making it difficult to interpret and compare results.

The application of economic methods to complex maternal quality improvement/health system interventions is more challenging than in other areas of health [39]. Cost-effectiveness includes measuring both cost and effectiveness. In the case of MPDSR, separate studies are required to assess reduction of maternal and perinatal mortality, which are logistically challenging and expensive. Only two such studies were identified by the Cochrane review on MPDSR, evaluating its impact on mortality reduction [14].

### Implications for policy and practice

Resources required to implement and maintain MPDSR will differ across system levels, populations, and countries. The maternal neonatal health mortality transition framework [40] may be able to serve as a guide for countries to assess their priorities for implementation based on the level of mortality within the context of their country. Likewise, WHO guidelines recommend countries use a phased approach for MPDSR implementation—start small and scale up [5, 7]. These guidelines allow for adaptability of system coverage because the implementation and maintenance mechanism will vary depending on the needs of a specific health system.

Notification and review of deaths at the facility level can be integrated into the existing roles of health workers to minimize the additional resources required to implement MPDSR [12]. This would also increase the skills and knowledge of the health workers, thereby helping to improve quality of care and implementation of other evidence-based interventions [12]. However, there is an opportunity cost in terms of health worker time spent on MPDSR activities, which may distract from other tasks—and that has not been properly measured or reported. Implementation of recommendations from the MPDSR process is also needed for the process to achieve its potential.

From this review, the most expensive part of MPDSR implementation was establishing a comprehensive surveillance system that identified all WRA deaths at the community level, especially if there is no pre-existing functional system of household surveillance [25, 32]. If a MDSR system is being set up, it is more cost-efficient to use the same system for reporting maternal, perinatal, and neonatal deaths, rather than maternal deaths alone [25, 32, 41, 42]. Similarly, data collection and review of maternal and perinatal deaths at the community level can be more cost-efficient if it is integrated into the routine roles of government health workers, including CHWs, rather than being implemented as a separate program. This approach may be more feasible in contexts with existing community health infrastructure [41, 42].

The quality of implementation of MPDSR plays an important role in determining whether it is effective [34]. The potential to save lives can occur only if the audit cycle is completed and recommendations are implemented over time, triggering iterative cycles of improvement [38, 43, 44]. As a complex intervention process, MPDSR must be considered part of a package of interventions that leads to strengthening the health system—such as quality improvement, leadership, and training—which will eventually enable mortality reduction [14], though there are many other factors beyond MDSPR that may influence mortality rates.

### Strengths and limitations

Despite our extensive search strategy, some relevant literature may have been missed. Our search strategy excluded: studies published prior to 2012, and/or set outside of a LMIC; editorials, commentaries, viewpoints, or essays; protocol- or abstract-only publications; and surveillance review articles. Our resulting dataset is small, including five papers representing four countries, which limited our analysis. The reported costs per death, costs per capita, and total costs, even after they were standardized to USD 2024 values, need to be interpreted with caution. The web-based tool (CCEMG-EPPI-Centre Cost Converter v.1.4 [ioe.ac.uk]) [26], which we used to convert costs to a common price year and currency, is a generic tool for use across a range of countries and sectors. As such, conversion rates based on purchasing power parities for gross domestic product are based on comparisons of prices for a sizeable and varied collection of goods and services that are not context-specific to health. Alternative context-specific approaches exist for healthcare but are more complex and dependent upon available data, which was beyond the scope of this review [45–48].

Despite these limitations, our review is comprehensive with the inclusion of grey literature and consultation with MPDSR subject matter experts, health economists, and the authors of included articles. The team met regularly to discuss our best analysis approach regarding the reported MPDSR implementation and maintenance costs. We contacted all authors of the included papers to obtain clarification on their methods and costs, but only two [24, 25] provided unpublished information on costs and contexts.

### Priorities for further research

Better data such as reporting cost categories, itemized costs, and changes in costs over the implementation period are needed for implementing MPDSR at different levels in the health system and improving context-specific recommendations. MPDSR does have implementation costs; however, integrating MPDSR with other surveillance systems may be cost-effective primarily for reducing maternal and perinatal mortality. There is debate about prioritizing implementation of MPDSR in resource-poor settings, versus investment in interventions to reduce deaths [35]. Economic evaluations reflecting the components of MPDSR can enable decision-making about implementation. More research is also needed to estimate costs of implementing recommendations, and to evaluate ways of integrating MPDSR into the health budgeting process. Evaluation tools such as the CHEERS checklist [28] and the Reference Case Guide [49] developed by the Global Health Cost Consortium may be used to further estimate and understand the costs of implementing MPDSR systems.

One study suggests that surveillance at the community level is more expensive than notifying and reviewing facility deaths, and it therefore requires more evidence regarding its cost–benefit [24]. Active death identification, through community surveys and investigation of all deaths of WRA, identifies many more deaths, but it is much more expensive than relying on passive reporting. The development of more sustainable, cost-efficient systems provides an opportunity to improve comprehensive reporting of maternal deaths, integrated within routine activities of government health workers, as has been done in Bangladesh [24].

Building the capacity in LMICs to enable surveillance system designers, operators, and users to understand the importance of cost estimates, and training them on consistent costing methods, can increase the completion and improve the quality of MPDSR economic evaluations. Developing evidence-based guidance for governments, researchers, and project managers on effectively estimating and reporting the economic value of MPDSR may be useful to advance programmatic and research efforts. To compare start-up and maintenance processes across health system levels, populations, and countries, costs can be reported in specific categories—such as those suggested in Table 5, and in Supplementary Tables 2 and 3a–3c.

## Conclusion

Based on the three studies in this review, setting up a new community-based surveillance system is more expensive than building on existing systems. However, the cost–benefit of community death surveillance and review, while more comprehensive because it has the potential to include all deaths, has not been clearly established. This review found no studies that document data on cost-effectiveness of MPDSR or incremental cost-effectiveness of different components of the intervention process. Only five studies were identified that described the MPDSR-related costs, from which only two studies [24, 25] had data in all cost categories used in this this study. Additional gaps in the literature include the MPDSR cost at different levels of the health system, the cost to governments, and the cost difference between maternal and perinatal deaths surveillance and review processes.

More research is needed on the most cost-effective approaches related to MPDSR in different contexts. The wide variation in economic methods, study designs, and contexts impeded direct comparison across studies. Standard MPDSR-related cost reporting can aid comprehensive analyses and comparisons in the future. Developing MPDSR-specific reporting guidelines, building skills, and demonstrating the value of economic methods for the development of sustainable MPDSR systems can help to address the challenges surrounding inconsistent economic methodologies and study comparisons.

## Supplementary Information

Supplementary Material 1

Supplementary Material 2

Supplementary Material 3

Supplementary Material 4

Supplementary Material 5

## Data Availability

The data described in this article can be freely and openly accessed from the papers included in our review [24, 25, 29–31] and our supplementary materials.

## Abbreviations

MPDSR: Maternal and perinatal death surveillance response
LMIC: Low- to middle-income country
USD: United States dollar
SDG: Sustainability Development Goals
WHO: World health organization
MDSR: Maternal death surveillance and response
PRISMA: Preferred reporting items for systematic reviews and meta-analyses
CHEERS: Consolidated health economic evaluation reporting standards
VA: Verbal autopsy
CHW: Community health worker
SMGL: Saving mothers giving life
RAMOS: Reproductive-age mortality study
VHT: Village health team
WRA: Women of reproductive age
