# Supplementary material for: Cost of implementation and maintenance of maternal and perinatal death surveillance and response: a scoping review
Source: BMC Pregnancy Childbirth. 2025 Oct 6;25(Suppl 1):1016. doi: 10.1186/s12884-025-08181-z (PMC12498452; doi:10.1186/s12884-025-08181-z)
Supplement: Supplementary file 1 — Supplementary Material 1 [file 12884_2025_8181_MOESM1_ESM.docx]

| Supplementary Table 1. Scoping review search strategy, by database, *n* = 11 | |  |
| --- | --- | --- |
| **Database** | **Strategy** |  |
| **Medline (OVID)** | Maternal Health/ OR Maternal Health Services/ OR Infant Health/ OR Maternal Mortality/ OR Fetal Mortality/ OR (maternal health OR neonatal health OR infant health OR newborn health OR maternal mortality OR infant mortality OR newborn mortality OR neonatal mortality OR maternal death* OR newborn death* OR neonatal death* OR infant death* OR antenatal care OR prenatal care OR perinatal care OR (safe* ADJ2 motherhood) OR maternity care OR perinatal death* OR perinatal mortality OR stillbirth* OR still birth* OR stillborn OR still born OR F?etal death OR F?etal demise).ti,ab,kf,hw. |  |
|  | AND |  |
|  | (Program Evaluation/ AND sustainable.ti,ab,kf,hw.) OR ((monitor* ADJ5 evaluat*) OR Sustainability OR (Sustainab* ADJ5 (evaluation OR monitor* OR index OR assess* OR doctrine* OR health* OR review OR framework OR strateg* OR program* OR intervention* OR development OR audit OR confidential inquiry OR surveillance)) OR localization OR scale-up).mp. |  |
|  | AND |  |
|  | exp "Costs and Cost Analysis"/ OR (Costs OR (cost ADJ2 analysis) OR cost benefit* OR economic*).mp. |  |
|  | AND |  |
|  | (afghan* OR africa* OR algeria* OR angola* OR bangladesh* OR benin* OR bhutan* OR bolivia* OR burkina* OR burkinese* OR burundi* OR urundi* OR cabo verde* OR cape verde* OR cambodia* OR cameroon* OR cameroun* OR chad* OR comoro* OR comore* OR comorian* OR congo* OR "cote d'ivoir*" OR "cote d' ivoir*" OR cote divoir* OR cote d ivoir* OR ivory coast* OR ivorian* OR Djibouti OR Egypt* OR el Salvador OR guinea* OR equatoguinea* OR Eritrea OR eswatini* OR swaziland* OR swazi* OR swati* OR ethiopia* OR gambia* OR ghana OR haiti* OR hondura* OR india* OR indonesia* OR iran* OR kenya* OR korea* OR laos OR lao OR laotian* OR lebanon* OR Lebanese OR Liberia OR madagasca OR malawi* OR mali OR Micronesia* OR mauritania* OR mauritan* OR Mauritius OR mongol* OR morocco* OR Moroccan* OR mozambique* OR mozambican* OR Myanmar OR Nepal OR nicaragua* OR niger* OR Pakistan OR gaza* OR west bank OR philippine* OR philipine* OR phillipine* OR phillippine* OR filipino* OR filipina OR rwanda* OR rwandese OR ruanda* OR ruandese OR samoa OR polynesia* OR "sao tome and principe*" OR senegal* OR sierra leone* OR solomon island OR somali* OR sri lanka OR sudan* OR Syria OR tajik* OR tanzania* OR tanganyika* OR timor leste* OR east timor* OR timorese* OR togo OR togoles* OR "togo's" OR tonga* OR tunisia* OR uganda* OR ukrain* OR uzbek* OR vanuatu * OR vietnam* OR viet nam* OR yemen* OR zambia* OR zimbabwe* OR southeastern asia* OR south eastern asia* OR southeast asia* OR south east asia* OR developing countr* OR developing nation* OR developing population* OR developing world OR less developed countr* OR less developed nation* OR less developed world OR lesser developed countr* OR lesser developed nation* OR lesser developed world OR under developed countr* OR under developed nation* OR under developed world OR underdeveloped countr* OR underdeveloped nation* OR underdeveloped world OR low income countr* OR low income nation* OR low income population* OR lower income countr* OR lower income nation* OR lower income population* OR underserved countr* OR underserved nation* OR underserved population* OR under served population* OR under served nation* OR under served population* OR deprived countr* OR deprived population* OR high burden countr* OR high burden nation* OR countdown countr* OR countdown nation* OR poor countr* OR poor nation* OR poor population* OR poor world OR poorer countr* OR poorer nation* OR poorer population* OR poorer world OR developing econom* OR less developed econom* OR underdeveloped econom* OR under developed econom*).ti,ab,hw,kf. |  |
|  | Limit by publication type journal article |  |
|  | Limit 2012 to present; |  |
| **Embase (OVID)** | Maternal welfare/ OR Maternal Health Service/ OR Maternal Mortality/ OR Fetus Mortality/ OR (maternal health OR neonatal health OR infant health OR newborn health OR maternal mortality OR infant mortality OR newborn mortality OR neonatal mortality OR maternal death* OR newborn death* OR neonatal death* OR infant death* OR antenatal care OR prenatal care OR perinatal care OR (safe* ADJ2 motherhood) OR maternity care OR perinatal death* OR perinatal mortality OR stillbirth* OR still birth* OR stillborn OR still born OR F?etal death OR F?etal demise).ti,ab,kf,hw. |  |
|  | AND |  |
|  | [(Program Evaluation/ AND sustainable.ti,ab,kf,hw.) OR ((monitor* ADJ5 evaluat*) OR Sustainability OR (Sustainab* ADJ5 (evaluation OR monitor* OR index OR assess* OR doctrine* OR health* OR review OR framework OR strateg* OR program* OR intervention* OR development OR audit or review) OR localization OR scale-up OR (cost ADJ2 analysis) OR costs OR economic*).ti,ab,kf,hw.](https://ovidsp.dc1.ovid.com/ovid-a/ovidweb.cgi?&S=IJBKFPJADLACOFKLKPMJFHPKNOHCAA00&Search+Link=%22Participatory+monitoring+and+evaluation%22.kw.) |  |
|  | AND |  |
|  | Exp Cost/ OR (Costs OR (cost ADJ2 analysis) OR cost benefit* OR economic*).mp. |  |
|  | AND |  |
|  | (afghan* OR africa* OR algeria* OR angola* OR bangladesh* OR benin* OR bhutan* OR bolivia* OR burkina* OR burkinese* OR burundi* OR urundi* OR cabo verde* OR cape verde* OR cambodia* OR cameroon* OR cameroun* OR chad* OR comoro* OR comore* OR comorian* OR congo* OR "cote d'ivoir*" OR "cote d' ivoir*" OR cote divoir* OR cote d ivoir* OR ivory coast* OR ivorian* OR Djibouti OR Egypt* OR el Salvador OR guinea* OR equatoguinea* OR Eritrea OR eswatini* OR swaziland* OR swazi* OR swati* OR ethiopia* OR gambia* OR ghana OR haiti* OR hondura* OR india* OR indonesia* OR iran* OR kenya* OR korea* OR laos OR lao OR laotian* OR lebanon* OR Lebanese OR Liberia OR madagasca OR malawi* OR mali OR Micronesia* OR mauritania* OR mauritan* OR Mauritius OR mongol* OR morocco* OR Moroccan* OR mozambique* OR mozambican* OR Myanmar OR Nepal OR nicaragua* OR niger* OR Pakistan OR gaza* OR west bank OR philippine* OR philipine* OR phillipine* OR phillippine* OR filipino* OR filipina OR rwanda* OR rwandese OR ruanda* OR ruandese OR samoa OR polynesia* OR "sao tome and principe*" OR senegal* OR sierra leone* OR solomon island OR somali* OR sri lanka OR sudan* OR Syria OR tajik* OR tanzania* OR tanganyika* OR timor leste* OR east timor* OR timorese* OR togo OR togoles* OR "togo's" OR tonga* OR tunisia* OR uganda* OR ukrain* OR uzbek* OR vanuatu * OR vietnam* OR viet nam* OR yemen* OR zambia* OR zimbabwe* OR southeastern asia* OR south eastern asia* OR southeast asia* OR south east asia* OR developing countr* OR developing nation* OR developing population* OR developing world OR less developed countr* OR less developed nation* OR less developed world OR lesser developed countr* OR lesser developed nation* OR lesser developed world OR under developed countr* OR under developed nation* OR under developed world OR underdeveloped countr* OR underdeveloped nation* OR underdeveloped world OR low income countr* OR low income nation* OR low income population* OR lower income countr* OR lower income nation* OR lower income population* OR underserved countr* OR underserved nation* OR underserved population* OR under served population* OR under served nation* OR under served population* OR deprived countr* OR deprived population* OR high burden countr* OR high burden nation* OR countdown countr* OR countdown nation* OR poor countr* OR poor nation* OR poor population* OR poor world OR poorer countr* OR poorer nation* OR poorer population* OR poorer world OR developing econom* OR less developed econom* OR underdeveloped econom* OR under developed econom*).ti,ab,hw,kf. |  |
|  | AND |  |
|  | Article.pt |  |
|  | Limit 2012 to present; |  |
|  | Remove Medline Records; NOT conference abstracts |  |
| **Global Health (OVID)** | (maternal health OR neonatal health OR infant health OR newborn health OR maternal mortality OR infant mortality OR newborn mortality OR neonatal mortality OR maternal death* OR newborn death* OR neonatal death* OR infant death* OR antenatal care OR prenatal care OR perinatal care OR (safe* ADJ2 motherhood) OR maternity care OR perinatal death* OR perinatal mortality OR stillbirth* OR still birth* OR stillborn OR still born OR F?etal death OR F?etal demise).ti,ab,sh,id. |  |
|  | AND |  |
|  | ((monitor* ADJ5 evaluat*) OR Sustainability OR (Sustainab* ADJ5 (evaluation OR monitor* OR index OR assess* OR doctrine* OR health* OR review OR framework OR strateg* OR program* OR intervention* OR development OR audit OR review OR confidential inquiry OR surveillance)) OR localization OR scale-up).mp. |  |
|  | AND |  |
|  | Cost.id,sh. OR (Costs OR (cost ADJ2 analysis) OR cost benefit* OR economic*).mp. |  |
|  | AND |  |
|  | (afghan* OR africa* OR algeria* OR angola* OR bangladesh* OR benin* OR bhutan* OR bolivia* OR burkina* OR burkinese* OR burundi* OR urundi* OR cabo verde* OR cape verde* OR cambodia* OR cameroon* OR cameroun* OR chad* OR comoro* OR comore* OR comorian* OR congo* OR "cote d'ivoir*" OR "cote d' ivoir*" OR cote divoir* OR cote d ivoir* OR ivory coast* OR ivorian* OR Djibouti OR Egypt* OR el Salvador OR guinea* OR equatoguinea* OR Eritrea OR eswatini* OR swaziland* OR swazi* OR swati* OR ethiopia* OR gambia* OR ghana OR haiti* OR hondura* OR india* OR indonesia* OR iran* OR kenya* OR korea* OR laos OR lao OR laotian* OR lebanon* OR Lebanese OR Liberia OR madagasca OR malawi* OR mali OR Micronesia* OR mauritania* OR mauritan* OR Mauritius OR mongol* OR morocco* OR Moroccan* OR mozambique* OR mozambican* OR Myanmar OR Nepal OR nicaragua* OR niger* OR Pakistan OR gaza* OR west bank OR philippine* OR philipine* OR phillipine* OR phillippine* OR filipino* OR filipina OR rwanda* OR rwandese OR ruanda* OR ruandese OR samoa OR polynesia* OR "sao tome and principe*" OR senegal* OR sierra leone* OR solomon island OR somali* OR sri lanka OR sudan* OR Syria OR tajik* OR tanzania* OR tanganyika* OR timor leste* OR east timor* OR timorese* OR togo OR togoles* OR "togo's" OR tonga* OR tunisia* OR uganda* OR ukrain* OR uzbek* OR vanuatu * OR vietnam* OR viet nam* OR yemen* OR zambia* OR zimbabwe* OR southeastern asia* OR south eastern asia* OR southeast asia* OR south east asia* OR developing countr* OR developing nation* OR developing population* OR developing world OR less developed countr* OR less developed nation* OR less developed world OR lesser developed countr* OR lesser developed nation* OR lesser developed world OR under developed countr* OR under developed nation* OR under developed world OR underdeveloped countr* OR underdeveloped nation* OR underdeveloped world OR low income countr* OR low income nation* OR low income population* OR lower income countr* OR lower income nation* OR lower income population* OR underserved countr* OR underserved nation* OR underserved population* OR under served population* OR under served nation* OR under served population* OR deprived countr* OR deprived population* OR high burden countr* OR high burden nation* OR countdown countr* OR countdown nation* OR poor countr* OR poor nation* OR poor population* OR poor world OR poorer countr* OR poorer nation* OR poorer population* OR poorer world OR developing econom* OR less developed econom* OR underdeveloped econom* OR under developed econom*).ti,ab,sh,id. |  |
|  | Limit 2012 to present; |  |
| **Cochrane Library** | [mh ^"Maternal Health"] OR [mh ^"Maternal Health Services"] OR [mh ^"Infant Health"] OR [mh ^"Maternal Mortality"] OR [mh ^"Fetal Mortality"] OR ("maternal health":ti,ab,kw OR "neonatal health":ti,ab,kw OR "infant health":ti,ab,kw OR "newborn health":ti,ab,kw OR "maternal mortality":ti,ab,kw OR "infant mortality":ti,ab,kw OR "newborn mortality":ti,ab,kw OR "neonatal mortality":ti,ab,kw OR (maternal NEXT death*):ti,ab,kw OR (newborn NEXT death*):ti,ab,kw OR (neonatal NEXT death*):ti,ab,kw OR (infant NEXT death*):ti,ab,kw OR "antenatal care":ti,ab,kw OR "prenatal care":ti,ab,kw OR "perinatal care":ti,ab,kw OR (safe*:ti,ab,kw NEAR/2 motherhood:ti,ab,kw) OR "maternity care":ti,ab,kw OR perinatal NEXT death*:ti,ab,kw OR "perinatal mortality":ti,ab,kw OR stillbirth*:ti,ab,kw OR still NEXT birth*:ti,ab,kw OR stillborn:ti,ab,kw OR "still born":ti,ab,kw OR "F?etal death":ti,ab,kw OR F?etal NEXT demise:ti,ab,kw) |  |
|  | AND |  |
|  | ([mh ^"Program Evaluation"] AND sustainable:ti,ab,kw) OR ((monitor*:ti,ab,kw NEAR/5 evaluat*:ti,ab,kw) OR Sustainability:ti,ab,kw OR (Sustainab*:ti,ab,kw NEAR/5 (evaluation:ti,ab,kw OR monitor*:ti,ab,kw OR index:ti,ab,kw OR assess*:ti,ab,kw OR doctrine*:ti,ab,kw OR health*:ti,ab,kw OR review:ti,ab,kw OR framework:ti,ab,kw OR strateg*:ti,ab,kw OR program*:ti,ab,kw OR intervention*:ti,ab,kw OR development:ti,ab,kw OR audit:ti,ab,kw OR review OR "confidential inquiry":ti,ab,kw OR surveillance:ti,ab,kw)) OR localization:ti,ab,kw OR scale-up:ti,ab,kw) |  |
|  | AND |  |
|  | (Costs OR (cost NEAR/2 analysis) OR ("cost" NEXT benefit*) OR economic*):ti,ab,kw |  |
|  | AND |  |
|  | (afghan*:ti,ab,kw OR africa*:ti,ab,kw OR algeria*:ti,ab,kw OR angola*:ti,ab,kw OR bangladesh*:ti,ab,kw OR benin*:ti,ab,kw OR bhutan*:ti,ab,kw OR bolivia*:ti,ab,kw OR burkina*:ti,ab,kw OR burkinese*:ti,ab,kw OR burundi*:ti,ab,kw OR urundi*:ti,ab,kw OR ("cabo" NEXT verde*):ti,ab,kw OR ("cape" NEXT verde*):ti,ab,kw OR cambodia*:ti,ab,kw OR cameroon*:ti,ab,kw OR cameroun*:ti,ab,kw OR chad*:ti,ab,kw OR comoro*:ti,ab,kw OR comore*:ti,ab,kw OR comorian*:ti,ab,kw OR congo*:ti,ab,kw OR ("cote" NEXT d?ivoir*):ti,ab,kw OR ("cote d?" NEXT ivoir*):ti,ab,kw OR ("cote" NEXT divoir*):ti,ab,kw OR ("cote d" NEXT ivoir*):ti,ab,kw OR ("ivory" NEXT coast*):ti,ab,kw OR ivorian*:ti,ab,kw OR Djibouti:ti,ab,kw OR Egypt*:ti,ab,kw OR "el Salvador":ti,ab,kw OR guinea*:ti,ab,kw OR equatoguinea*:ti,ab,kw OR Eritrea:ti,ab,kw OR eswatini*:ti,ab,kw OR swaziland*:ti,ab,kw OR swazi*:ti,ab,kw OR swati*:ti,ab,kw OR ethiopia*:ti,ab,kw OR gambia*:ti,ab,kw OR ghana:ti,ab,kw OR haiti*:ti,ab,kw OR hondura*:ti,ab,kw OR india*:ti,ab,kw OR indonesia*:ti,ab,kw OR iran*:ti,ab,kw OR kenya*:ti,ab,kw OR korea*:ti,ab,kw OR laos:ti,ab,kw OR lao:ti,ab,kw OR laotian*:ti,ab,kw OR lebanon*:ti,ab,kw OR Lebanese:ti,ab,kw OR Liberia:ti,ab,kw OR madagasca:ti,ab,kw OR malawi*:ti,ab,kw OR mali:ti,ab,kw OR Micronesia*:ti,ab,kw OR mauritania*:ti,ab,kw OR mauritan*:ti,ab,kw OR Mauritius:ti,ab,kw OR mongol*:ti,ab,kw OR morocco*:ti,ab,kw OR Moroccan*:ti,ab,kw OR mozambique*:ti,ab,kw OR mozambican*:ti,ab,kw OR Myanmar:ti,ab,kw OR Nepal:ti,ab,kw OR nicaragua*:ti,ab,kw OR niger*:ti,ab,kw OR Pakistan:ti,ab,kw OR gaza*:ti,ab,kw OR "west bank":ti,ab,kw OR philippine*:ti,ab,kw OR philipine*:ti,ab,kw OR phillipine*:ti,ab,kw OR phillippine*:ti,ab,kw OR filipino*:ti,ab,kw OR filipina:ti,ab,kw OR rwanda*:ti,ab,kw OR rwandese:ti,ab,kw OR ruanda*:ti,ab,kw OR ruandese:ti,ab,kw OR samoa:ti,ab,kw OR polynesia*:ti,ab,kw OR ("sao tome" NEXT principe*):ti,ab,kw OR senegal*:ti,ab,kw OR ("sierra" NEXT leone*):ti,ab,kw OR "solomon island":ti,ab,kw OR somali*:ti,ab,kw OR "sri lanka":ti,ab,kw OR sudan*:ti,ab,kw OR Syria:ti,ab,kw OR tajik*:ti,ab,kw OR tanzania*:ti,ab,kw OR tanganyika*:ti,ab,kw OR ("timor" NEXT leste*):ti,ab,kw OR ("east" NEXT timor*):ti,ab,kw OR timorese*:ti,ab,kw OR togo:ti,ab,kw OR togoles*:ti,ab,kw OR togo:ti,ab,kw OR tonga*:ti,ab,kw OR tunisia*:ti,ab,kw OR uganda*:ti,ab,kw OR ukrain*:ti,ab,kw OR uzbek*:ti,ab,kw OR ("vanuatu" NEXT *):ti,ab,kw OR vietnam*:ti,ab,kw OR ("viet" NEXT nam*):ti,ab,kw OR yemen*:ti,ab,kw OR zambia*:ti,ab,kw OR zimbabwe*:ti,ab,kw OR ("southeastern" NEXT asia*):ti,ab,kw OR ("south eastern" NEXT asia*):ti,ab,kw OR ("southeast" NEXT asia*):ti,ab,kw OR ("south east" NEXT asia*):ti,ab,kw OR ("developing" NEXT countr*):ti,ab,kw OR ("developing" NEXT nation*):ti,ab,kw OR ("developing" NEXT population*):ti,ab,kw OR "developing world":ti,ab,kw OR ("less developed" NEXT countr*):ti,ab,kw OR ("less developed" NEXT nation*):ti,ab,kw OR "less developed world":ti,ab,kw OR ("lesser developed" NEXT countr*):ti,ab,kw OR ("lesser developed" NEXT nation*):ti,ab,kw OR "lesser developed world":ti,ab,kw OR ("under developed" NEXT countr*):ti,ab,kw OR ("under developed" NEXT nation*):ti,ab,kw OR "under developed world":ti,ab,kw OR ("underdeveloped" NEXT countr*):ti,ab,kw OR ("underdeveloped" NEXT nation*):ti,ab,kw OR "underdeveloped world":ti,ab,kw OR ("low income" NEXT countr*):ti,ab,kw OR ("low income" NEXT nation*):ti,ab,kw OR ("low income" NEXT population*):ti,ab,kw OR ("lower income" NEXT countr*):ti,ab,kw OR ("lower income" NEXT nation*):ti,ab,kw OR ("lower income" NEXT population*):ti,ab,kw OR ("underserved" NEXT countr*):ti,ab,kw OR ("underserved" NEXT nation*):ti,ab,kw OR ("underserved" NEXT population*):ti,ab,kw OR ("under served" NEXT population*):ti,ab,kw OR ("under served" NEXT nation*):ti,ab,kw OR ("under served" NEXT population*):ti,ab,kw OR ("deprived" NEXT countr*):ti,ab,kw OR ("deprived" NEXT population*):ti,ab,kw OR ("high burden" NEXT countr*):ti,ab,kw OR ("high burden" NEXT nation*):ti,ab,kw OR ("countdown" NEXT countr*):ti,ab,kw OR ("countdown" NEXT nation*):ti,ab,kw OR ("poor" NEXT countr*):ti,ab,kw OR ("poor" NEXT nation*):ti,ab,kw OR ("poor" NEXT population*):ti,ab,kw OR "poor world":ti,ab,kw OR ("poorer" NEXT countr*):ti,ab,kw OR ("poorer" NEXT nation*):ti,ab,kw OR ("poorer" NEXT population*):ti,ab,kw OR "poorer world":ti,ab,kw OR ("developing" NEXT econom*):ti,ab,kw OR ("less developed" NEXT econom*):ti,ab,kw OR ("underdeveloped" NEXT econom*):ti,ab,kw OR ("under developed" NEXT econom*):ti,ab,kw) |  |
|  | 2012 - 2023 |  |
| **CINAHL (EbscoHost)** | (MH "Maternal Health") OR (MH "Maternal Health Services") OR (MH "Infant Health") OR (MH "Maternal Mortality") OR (MH "Fetal Mortality") OR ((TI "maternal health" OR AB "maternal health" OR SU "maternal health") OR (TI "neonatal health" OR AB "neonatal health" OR SU "neonatal health") OR (TI "infant health" OR AB "infant health" OR SU "infant health") OR (TI "newborn health" OR AB "newborn health" OR SU "newborn health") OR (TI "maternal mortality" OR AB "maternal mortality" OR SU "maternal mortality") OR (TI "infant mortality" OR AB "infant mortality" OR SU "infant mortality") OR (TI "newborn mortality" OR AB "newborn mortality" OR SU "newborn mortality") OR (TI "neonatal mortality" OR AB "neonatal mortality" OR SU "neonatal mortality") OR (TI "maternal death*" OR AB "maternal death*" OR SU "maternal death*") OR (TI "newborn death*" OR AB "newborn death*" OR SU "newborn death*") OR (TI "neonatal death*" OR AB "neonatal death*" OR SU "neonatal death*") OR (TI "infant death*" OR AB "infant death*" OR SU "infant death*") OR (TI "antenatal care" OR AB "antenatal care" OR SU "antenatal care") OR (TI "prenatal care" OR AB "prenatal care" OR SU "prenatal care") OR (TI "perinatal care" OR AB "perinatal care" OR SU "perinatal care") OR ((TI safe* OR AB safe* OR SU safe*) N2 (TI motherhood OR AB motherhood OR SU motherhood)) OR (TI "maternity care" OR AB "maternity care" OR SU "maternity care") OR ((TI "perinatal death*" OR AB "perinatal death*" OR SU "perinatal death*") OR (TI "perinatal mortality" OR AB "perinatal mortality" OR SU "perinatal mortality") OR (TI stillbirth* OR AB stillbirth* OR SU stillbirth*) OR (TI "still birth*" OR AB "still birth*" OR SU "still birth*") OR (TI stillborn OR AB stillborn OR SU stillborn) OR (TI "still born" OR AB "still born" OR SU "still born") OR (TI "F#etal death" OR AB "F#etal death" OR SU "F#etal death") OR (TI "F#etal demise" OR AB "F#etal demise" OR SU "F#etal demise")) |  |
|  | AND |  |
|  | ((MH "Program Evaluation") AND sustainable) OR ((monitor* N5 evaluat*) OR Sustainability OR (Sustainab* N5 (evaluation OR monitor* OR index OR assess* OR doctrine* OR health* OR review OR framework OR strateg* OR program* OR intervention* OR development OR audit OR "confidential inquiry" OR surveillance)) OR localization OR scale-up) |  |
|  | AND |  |
|  | (TI (Costs OR (cost N2 analysis) OR "cost benefit*" OR economic*)) OR (AB (Costs OR (cost N2 analysis) OR "cost benefit*" OR economic*)) OR (SU (Costs OR (cost N2 analysis) OR "cost benefit*" OR economic*)) |  |
|  | AND |  |
|  | ((TI afghan* OR AB afghan* OR SU afghan*) OR (TI africa* OR AB africa* OR SU africa*) OR (TI algeria* OR AB algeria* OR SU algeria*) OR (TI angola* OR AB angola* OR SU angola*) OR (TI bangladesh* OR AB bangladesh* OR SU bangladesh*) OR (TI benin* OR AB benin* OR SU benin*) OR (TI bhutan* OR AB bhutan* OR SU bhutan*) OR (TI bolivia* OR AB bolivia* OR SU bolivia*) OR (TI burkina* OR AB burkina* OR SU burkina*) OR (TI burkinese* OR AB burkinese* OR SU burkinese*) OR (TI burundi* OR AB burundi* OR SU burundi*) OR (TI urundi* OR AB urundi* OR SU urundi*) OR (TI "cabo verde*" OR AB "cabo verde*" OR SU "cabo verde*") OR (TI "cape verde*" OR AB "cape verde*" OR SU "cape verde*") OR (TI cambodia* OR AB cambodia* OR SU cambodia*) OR (TI cameroon* OR AB cameroon* OR SU cameroon*) OR (TI cameroun* OR AB cameroun* OR SU cameroun*) OR (TI chad* OR AB chad* OR SU chad*) OR (TI comoro* OR AB comoro* OR SU comoro*) OR (TI comore* OR AB comore* OR SU comore*) OR (TI comorian* OR AB comorian* OR SU comorian*) OR (TI congo* OR AB congo* OR SU congo*) OR (TI "cote d'ivoir*" OR AB "cote d'ivoir*" OR SU "cote d'ivoir*") OR (TI "cote d' ivoir*" OR AB "cote d' ivoir*" OR SU "cote d' ivoir*") OR (TI "cote divoir*" OR AB "cote divoir*" OR SU "cote divoir*") OR (TI "cote d ivoir*" OR AB "cote d ivoir*" OR SU "cote d ivoir*") OR (TI "ivory coast*" OR AB "ivory coast*" OR SU "ivory coast*") OR (TI ivorian* OR AB ivorian* OR SU ivorian*) OR (TI Djibouti OR AB Djibouti OR SU Djibouti) OR (TI Egypt* OR AB Egypt* OR SU Egypt*) OR (TI "el Salvador" OR AB "el Salvador" OR SU "el Salvador") OR (TI guinea* OR AB guinea* OR SU guinea*) OR (TI equatoguinea* OR AB equatoguinea* OR SU equatoguinea*) OR (TI Eritrea OR AB Eritrea OR SU Eritrea) OR (TI eswatini* OR AB eswatini* OR SU eswatini*) OR (TI swaziland* OR AB swaziland* OR SU swaziland*) OR (TI swazi* OR AB swazi* OR SU swazi*) OR (TI swati* OR AB swati* OR SU swati*) OR (TI ethiopia* OR AB ethiopia* OR SU ethiopia*) OR (TI gambia* OR AB gambia* OR SU gambia*) OR (TI ghana OR AB ghana OR SU ghana) OR (TI haiti* OR AB haiti* OR SU haiti*) OR (TI hondura* OR AB hondura* OR SU hondura*) OR (TI india* OR AB india* OR SU india*) OR (TI indonesia* OR AB indonesia* OR SU indonesia*) OR (TI iran* OR AB iran* OR SU iran*) OR (TI kenya* OR AB kenya* OR SU kenya*) OR (TI korea* OR AB korea* OR SU korea*) OR (TI laos OR AB laos OR SU laos) OR (TI lao OR AB lao OR SU lao) OR (TI laotian* OR AB laotian* OR SU laotian*) OR (TI lebanon* OR AB lebanon* OR SU lebanon*) OR (TI Lebanese OR AB Lebanese OR SU Lebanese) OR (TI Liberia OR AB Liberia OR SU Liberia) OR (TI madagasca OR AB madagasca OR SU madagasca) OR (TI malawi* OR AB malawi* OR SU malawi*) OR (TI mali OR AB mali OR SU mali) OR (TI Micronesia* OR AB Micronesia* OR SU Micronesia*) OR (TI mauritania* OR AB mauritania* OR SU mauritania*) OR (TI mauritan* OR AB mauritan* OR SU mauritan*) OR (TI Mauritius OR AB Mauritius OR SU Mauritius) OR (TI mongol* OR AB mongol* OR SU mongol*) OR (TI morocco* OR AB morocco* OR SU morocco*) OR (TI Moroccan* OR AB Moroccan* OR SU Moroccan*) OR (TI mozambique* OR AB mozambique* OR SU mozambique*) OR (TI mozambican* OR AB mozambican* OR SU mozambican*) OR (TI Myanmar OR AB Myanmar OR SU Myanmar) OR (TI Nepal OR AB Nepal OR SU Nepal) OR (TI nicaragua* OR AB nicaragua* OR SU nicaragua*) OR (TI niger* OR AB niger* OR SU niger*) OR (TI Pakistan OR AB Pakistan OR SU Pakistan) OR (TI gaza* OR AB gaza* OR SU gaza*) OR (TI "west bank" OR AB "west bank" OR SU "west bank") OR (TI philippine* OR AB philippine* OR SU philippine*) OR (TI philipine* OR AB philipine* OR SU philipine*) OR (TI phillipine* OR AB phillipine* OR SU phillipine*) OR (TI phillippine* OR AB phillippine* OR SU phillippine*) OR (TI filipino* OR AB filipino* OR SU filipino*) OR (TI filipina OR AB filipina OR SU filipina) OR (TI rwanda* OR AB rwanda* OR SU rwanda*) OR (TI rwandese OR AB rwandese OR SU rwandese) OR (TI ruanda* OR AB ruanda* OR SU ruanda*) OR (TI ruandese OR AB ruandese OR SU ruandese) OR (TI samoa OR AB samoa OR SU samoa) OR (TI polynesia* OR AB polynesia* OR SU polynesia*) OR (TI "sao tome and principe*" OR AB "sao tome and principe*" OR SU "sao tome and principe*") OR (TI senegal* OR AB senegal* OR SU senegal*) OR (TI "sierra leone*" OR AB "sierra leone*" OR SU "sierra leone*") OR (TI "solomon island" OR AB "solomon island" OR SU "solomon island") OR (TI somali* OR AB somali* OR SU somali*) OR (TI "sri lanka" OR AB "sri lanka" OR SU "sri lanka") OR (TI sudan* OR AB sudan* OR SU sudan*) OR (TI Syria OR AB Syria OR SU Syria) OR (TI tajik* OR AB tajik* OR SU tajik*) OR (TI tanzania* OR AB tanzania* OR SU tanzania*) OR (TI tanganyika* OR AB tanganyika* OR SU tanganyika*) OR (TI "timor leste*" OR AB "timor leste*" OR SU "timor leste*") OR (TI "east timor*" OR AB "east timor*" OR SU "east timor*") OR (TI timorese* OR AB timorese* OR SU timorese*) OR (TI togo OR AB togo OR SU togo) OR (TI togoles* OR AB togoles* OR SU togoles*) OR (TI togo's OR AB togo's OR SU togo's) OR (TI tonga* OR AB tonga* OR SU tonga*) OR (TI tunisia* OR AB tunisia* OR SU tunisia*) OR (TI uganda* OR AB uganda* OR SU uganda*) OR (TI ukrain* OR AB ukrain* OR SU ukrain*) OR (TI uzbek* OR AB uzbek* OR SU uzbek*) OR (TI "vanuatu *" OR AB "vanuatu *" OR SU "vanuatu *") OR (TI vietnam* OR AB vietnam* OR SU vietnam*) OR (TI "viet nam*" OR AB "viet nam*" OR SU "viet nam*") OR (TI yemen* OR AB yemen* OR SU yemen*) OR (TI zambia* OR AB zambia* OR SU zambia*) OR (TI zimbabwe* OR AB zimbabwe* OR SU zimbabwe*) OR (TI "southeastern asia*" OR AB "southeastern asia*" OR SU "southeastern asia*") OR (TI "south eastern asia*" OR AB "south eastern asia*" OR SU "south eastern asia*") OR (TI "southeast asia*" OR AB "southeast asia*" OR SU "southeast asia*") OR (TI "south east asia*" OR AB "south east asia*" OR SU "south east asia*") OR (TI "developing countr*" OR AB "developing countr*" OR SU "developing countr*") OR (TI "developing nation*" OR AB "developing nation*" OR SU "developing nation*") OR (TI "developing population*" OR AB "developing population*" OR SU "developing population*") OR (TI "developing world" OR AB "developing world" OR SU "developing world") OR (TI "less developed countr*" OR AB "less developed countr*" OR SU "less developed countr*") OR (TI "less developed nation*" OR AB "less developed nation*" OR SU "less developed nation*") OR (TI "less developed world" OR AB "less developed world" OR SU "less developed world") OR (TI "lesser developed countr*" OR AB "lesser developed countr*" OR SU "lesser developed countr*") OR (TI "lesser developed nation*" OR AB "lesser developed nation*" OR SU "lesser developed nation*") OR (TI "lesser developed world" OR AB "lesser developed world" OR SU "lesser developed world") OR (TI "under developed countr*" OR AB "under developed countr*" OR SU "under developed countr*") OR (TI "under developed nation*" OR AB "under developed nation*" OR SU "under developed nation*") OR (TI "under developed world" OR AB "under developed world" OR SU "under developed world") OR (TI "underdeveloped countr*" OR AB "underdeveloped countr*" OR SU "underdeveloped countr*") OR (TI "underdeveloped nation*" OR AB "underdeveloped nation*" OR SU "underdeveloped nation*") OR (TI "underdeveloped world" OR AB "underdeveloped world" OR SU "underdeveloped world") OR (TI "low income countr*" OR AB "low income countr*" OR SU "low income countr*") OR (TI "low income nation*" OR AB "low income nation*" OR SU "low income nation*") OR (TI "low income population*" OR AB "low income population*" OR SU "low income population*") OR (TI "lower income countr*" OR AB "lower income countr*" OR SU "lower income countr*") OR (TI "lower income nation*" OR AB "lower income nation*" OR SU "lower income nation*") OR (TI "lower income population*" OR AB "lower income population*" OR SU "lower income population*") OR (TI "underserved countr*" OR AB "underserved countr*" OR SU "underserved countr*") OR (TI "underserved nation*" OR AB "underserved nation*" OR SU "underserved nation*") OR (TI "underserved population*" OR AB "underserved population*" OR SU "underserved population*") OR (TI "under served population*" OR AB "under served population*" OR SU "under served population*") OR (TI "under served nation*" OR AB "under served nation*" OR SU "under served nation*") OR (TI "under served population*" OR AB "under served population*" OR SU "under served population*") OR (TI "deprived countr*" OR AB "deprived countr*" OR SU "deprived countr*") OR (TI "deprived population*" OR AB "deprived population*" OR SU "deprived population*") OR (TI "high burden countr*" OR AB "high burden countr*" OR SU "high burden countr*") OR (TI "high burden nation*" OR AB "high burden nation*" OR SU "high burden nation*") OR (TI "countdown countr*" OR AB "countdown countr*" OR SU "countdown countr*") OR (TI "countdown nation*" OR AB "countdown nation*" OR SU "countdown nation*") OR (TI "poor countr*" OR AB "poor countr*" OR SU "poor countr*") OR (TI "poor nation*" OR AB "poor nation*" OR SU "poor nation*") OR (TI "poor population*" OR AB "poor population*" OR SU "poor population*") OR (TI "poor world" OR AB "poor world" OR SU "poor world") OR (TI "poorer countr*" OR AB "poorer countr*" OR SU "poorer countr*") OR (TI "poorer nation*" OR AB "poorer nation*" OR SU "poorer nation*") OR (TI "poorer population*" OR AB "poorer population*" OR SU "poorer population*") OR (TI "poorer world" OR AB "poorer world" OR SU "poorer world") OR (TI "developing econom*" OR AB "developing econom*" OR SU "developing econom*") OR (TI "less developed econom*" OR AB "less developed econom*" OR SU "less developed econom*") OR (TI "underdeveloped econom*" OR AB "underdeveloped econom*" OR SU "underdeveloped econom*") OR (TI "under developed econom*" OR AB "under developed econom*" OR SU "under developed econom*")) |  |
|  |  |  |
|  |  |  |
|  |  |  |
|  | 2012 – 2023 ; exclude Medline records |  |
| **Scopus** | INDEXTERMS("Maternal Health") OR INDEXTERMS("Maternal Health Services") OR INDEXTERMS("Infant Health") OR INDEXTERMS("Maternal Mortality") OR INDEXTERMS("Fetal Mortality") OR TITLE-ABS-KEY("maternal health" OR "neonatal health" OR "infant health" OR "newborn health" OR "maternal mortality" OR "infant mortality" OR "newborn mortality" OR "neonatal mortality" OR "maternal death*" OR "newborn death*" OR "neonatal death*" OR "infant death*" OR "antenatal care" OR "prenatal care" OR "perinatal care" OR (safe* W/2 motherhood) OR "maternity care" OR "perinatal death*" OR "perinatal mortality" OR stillbirth* OR "still birth*" OR stillborn OR "still born" OR "F*etal death" OR "F*etal demise") |  |
|  | AND |  |
|  | (INDEXTERMS("Program Evaluation") AND TITLE-ABS-KEY(sustainable)) OR TITLE-ABS-KEY((monitor* W/5 evaluat*) OR Sustainability OR (Sustainab* W/5 (evaluation OR monitor* OR index OR assess* OR doctrine* OR health* OR review OR framework OR strateg* OR program* OR intervention* OR development OR audit OR "confidential inquiry" OR surveillance)) OR localization OR scale-up) |  |
|  | AND |  |
|  | TITLE-ABS-KEY(Costs OR (cost W/2 analysis) OR "cost benefit*" OR economic*) |  |
|  | AND |  |
|  | TITLE-ABS-KEY(afghan* OR africa* OR algeria* OR angola* OR bangladesh* OR benin* OR bhutan* OR bolivia* OR burkina* OR burkinese* OR burundi* OR urundi* OR "cabo verde*" OR "cape verde*" OR cambodia* OR cameroon* OR cameroun* OR chad* OR comoro* OR comore* OR comorian* OR congo* OR "cote d'ivoir*" OR "cote d' ivoir*" OR "cote divoir*" OR "cote d ivoir*" OR "ivory coast*" OR ivorian* OR Djibouti OR Egypt* OR "el Salvador" OR guinea* OR equatoguinea* OR Eritrea OR eswatini* OR swaziland* OR swazi* OR swati* OR ethiopia* OR gambia* OR ghana OR haiti* OR hondura* OR india* OR indonesia* OR iran* OR kenya* OR korea* OR laos OR lao OR laotian* OR lebanon* OR Lebanese OR Liberia OR madagasca OR malawi* OR mali OR Micronesia* OR mauritania* OR mauritan* OR Mauritius OR mongol* OR morocco* OR Moroccan* OR mozambique* OR mozambican* OR Myanmar OR Nepal OR nicaragua* OR niger* OR Pakistan OR gaza* OR "west bank" OR philippine* OR philipine* OR phillipine* OR phillippine* OR filipino* OR filipina OR rwanda* OR rwandese OR ruanda* OR ruandese OR samoa OR polynesia* OR "sao tome and principe*" OR senegal* OR "sierra leone*" OR "solomon island" OR somali* OR "sri lanka" OR sudan* OR Syria OR tajik* OR tanzania* OR tanganyika* OR "timor leste*" OR "east timor*" OR timorese* OR togo OR togoles* OR togo's OR tonga* OR tunisia* OR uganda* OR ukrain* OR uzbek* OR "vanuatu *" OR vietnam* OR "viet nam*" OR yemen* OR zambia* OR zimbabwe* OR "southeastern asia*" OR "south eastern asia*" OR "southeast asia*" OR "south east asia*" OR "developing countr*" OR "developing nation*" OR "developing population*" OR "developing world" OR "less developed countr*" OR "less developed nation*" OR "less developed world" OR "lesser developed countr*" OR "lesser developed nation*" OR "lesser developed world" OR "under developed countr*" OR "under developed nation*" OR "under developed world" OR "underdeveloped countr*" OR "underdeveloped nation*" OR "underdeveloped world" OR "low income countr*" OR "low income nation*" OR "low income population*" OR "lower income countr*" OR "lower income nation*" OR "lower income population*" OR "underserved countr*" OR "underserved nation*" OR "underserved population*" OR "under served population*" OR "under served nation*" OR "under served population*" OR "deprived countr*" OR "deprived population*" OR "high burden countr*" OR "high burden nation*" OR "countdown countr*" OR "countdown nation*" OR "poor countr*" OR "poor nation*" OR "poor population*" OR "poor world" OR "poorer countr*" OR "poorer nation*" OR "poorer population*" OR "poorer world" OR "developing econom*" OR "less developed econom*" OR "underdeveloped econom*" OR "under developed econom*") |  |
|  | AND |  |
|  | NOT INDEX(medline) |  |
|  | 2012 – 2023 |  |
| **ProQuest Dissertations and Theses** | TI,AB("maternal health" OR "neonatal health" OR "infant health" OR "newborn health" OR "maternal mortality" OR "infant mortality" OR "newborn mortality" OR "neonatal mortality" OR "maternal death*" OR "newborn death*" OR "neonatal death*" OR "infant death*" OR "antenatal care" OR "prenatal care" OR "perinatal care" OR (safe* NEAR/2 motherhood) OR "maternity care" OR "perinatal death*" OR "perinatal mortality" OR stillbirth* OR "still birth*" OR stillborn OR "still born" OR "F*etal death" OR "F*etal demise") |  |
|  | AND |  |
|  | (MESH.EXACT("Program Evaluation") AND TI,AB,IF(sustainable)) OR ((TI,AB,IF(monitor*) NEAR/5 TI,AB,IF(evaluat*)) OR TI,AB,IF(Sustainability) OR (TI,AB,IF(Sustainab*) NEAR/5 (TI,AB,IF(evaluation) OR TI,AB,IF(monitor*) OR TI,AB,IF(index) OR TI,AB,IF(assess*) OR TI,AB,IF(doctrine*) OR TI,AB,IF(health*) OR TI,AB,IF(review) OR TI,AB,IF(framework) OR TI,AB,IF(strateg*) OR TI,AB,IF(program*) OR TI,AB,IF(intervention*) OR TI,AB,IF(development) OR TI,AB,IF(audit) OR TI,AB,IF("confidential inquiry") OR TI,AB,IF(surveillance))) OR TI,AB,IF(localization) OR TI,AB,IF(scale-up)) |  |
|  | AND |  |
|  | (TI,AB,IF(Costs) OR (TI,AB,IF(cost) NEAR/2 TI,AB,IF(analysis)) OR TI,AB,IF("cost benefit*") OR TI,AB,IF(economic*)) |  |
|  | AND |  |
|  | TI,AB(afghan* OR africa* OR algeria* OR angola* OR bangladesh* OR benin* OR bhutan* OR bolivia* OR burkina* OR burkinese* OR burundi* OR urundi* OR "cabo verde*" OR "cape verde*" OR cambodia* OR cameroon* OR cameroun* OR chad* OR comoro* OR comore* OR comorian* OR congo* OR "cote d'ivoir*" OR "cote d' ivoir*" OR "cote divoir*" OR "cote d ivoir*" OR "ivory coast*" OR ivorian* OR Djibouti OR Egypt* OR "el Salvador" OR guinea* OR equatoguinea* OR Eritrea OR eswatini* OR swaziland* OR swazi* OR swati* OR ethiopia* OR gambia* OR ghana OR haiti* OR hondura* OR india* OR indonesia* OR iran* OR kenya* OR korea* OR laos OR lao OR laotian* OR lebanon* OR Lebanese OR Liberia OR madagasca OR malawi* OR mali OR Micronesia* OR mauritania* OR mauritan* OR Mauritius OR mongol* OR morocco* OR Moroccan* OR mozambique* OR mozambican* OR Myanmar OR Nepal OR nicaragua* OR niger* OR Pakistan OR gaza* OR "west bank" OR philippine* OR philipine* OR phillipine* OR phillippine* OR filipino* OR filipina OR rwanda* OR rwandese OR ruanda* OR ruandese OR samoa OR polynesia* OR "sao tome and principe*" OR senegal* OR "sierra leone*" OR "solomon island" OR somali* OR "sri lanka" OR sudan* OR Syria OR tajik* OR tanzania* OR tanganyika* OR "timor leste*" OR "east timor*" OR timorese* OR togo OR togoles* OR togo's OR tonga* OR tunisia* OR uganda* OR ukrain* OR uzbek* OR "vanuatu *" OR vietnam* OR "viet nam*" OR yemen* OR zambia* OR zimbabwe* OR "southeastern asia*" OR "south eastern asia*" OR "southeast asia*" OR "south east asia*" OR "developing countr*" OR "developing nation*" OR "developing population*" OR "developing world" OR "less developed countr*" OR "less developed nation*" OR "less developed world" OR "lesser developed countr*" OR "lesser developed nation*" OR "lesser developed world" OR "under developed countr*" OR "under developed nation*" OR "under developed world" OR "underdeveloped countr*" OR "underdeveloped nation*" OR "underdeveloped world" OR "low income countr*" OR "low income nation*" OR "low income population*" OR "lower income countr*" OR "lower income nation*" OR "lower income population*" OR "underserved countr*" OR "underserved nation*" OR "underserved population*" OR "under served population*" OR "under served nation*" OR "under served population*" OR "deprived countr*" OR "deprived population*" OR "high burden countr*" OR "high burden nation*" OR "countdown countr*" OR "countdown nation*" OR "poor countr*" OR "poor nation*" OR "poor population*" OR "poor world" OR "poorer countr*" OR "poorer nation*" OR "poorer population*" OR "poorer world" OR "developing econom*" OR "less developed econom*" OR "underdeveloped econom*" OR "under developed econom*") |  |
|  |  |  |
| **OpenGrey** | TI,AB("maternal health" OR "neonatal health" OR "infant health" OR "newborn health" OR "maternal mortality" OR "infant mortality" OR "newborn mortality" OR "neonatal mortality" OR "maternal death*" OR "newborn death*" OR "neonatal death*" OR "infant death*" OR "antenatal care" OR "prenatal care" OR "perinatal care" OR (safe* NEAR/2 motherhood) OR "maternity care" OR "perinatal death*" OR "perinatal mortality" OR stillbirth* OR "still birth*" OR stillborn OR "still born" OR "F*etal death" OR "F*etal demise") |  |
|  | AND |  |
|  | (MESH.EXACT("Program Evaluation") AND TI,AB,IF(sustainable)) OR ((TI,AB,IF(monitor*) NEAR/5 TI,AB,IF(evaluat*)) OR TI,AB,IF(Sustainability) OR (TI,AB,IF(Sustainab*) NEAR/5 (TI,AB,IF(evaluation) OR TI,AB,IF(monitor*) OR TI,AB,IF(index) OR TI,AB,IF(assess*) OR TI,AB,IF(doctrine*) OR TI,AB,IF(health*) OR TI,AB,IF(review) OR TI,AB,IF(framework) OR TI,AB,IF(strateg*) OR TI,AB,IF(program*) OR TI,AB,IF(intervention*) OR TI,AB,IF(development) OR TI,AB,IF(audit) OR TI,AB,IF("confidential inquiry") OR TI,AB,IF(surveillance))) OR TI,AB,IF(localization) OR TI,AB,IF(scale-up)) |  |
|  | AND |  |
|  | (TI,AB,IF(Costs) OR (TI,AB,IF(cost) NEAR/2 TI,AB,IF(analysis)) OR TI,AB,IF("cost benefit*") OR TI,AB,IF(economic*)) |  |
|  | AND |  |
|  | TI,AB(afghan* OR africa* OR algeria* OR angola* OR bangladesh* OR benin* OR bhutan* OR bolivia* OR burkina* OR burkinese* OR burundi* OR urundi* OR "cabo verde*" OR "cape verde*" OR cambodia* OR cameroon* OR cameroun* OR chad* OR comoro* OR comore* OR comorian* OR congo* OR "cote d'ivoir*" OR "cote d' ivoir*" OR "cote divoir*" OR "cote d ivoir*" OR "ivory coast*" OR ivorian* OR Djibouti OR Egypt* OR "el Salvador" OR guinea* OR equatoguinea* OR Eritrea OR eswatini* OR swaziland* OR swazi* OR swati* OR ethiopia* OR gambia* OR ghana OR haiti* OR hondura* OR india* OR indonesia* OR iran* OR kenya* OR korea* OR laos OR lao OR laotian* OR lebanon* OR Lebanese OR Liberia OR madagasca OR malawi* OR mali OR Micronesia* OR mauritania* OR mauritan* OR Mauritius OR mongol* OR morocco* OR Moroccan* OR mozambique* OR mozambican* OR Myanmar OR Nepal OR nicaragua* OR niger* OR Pakistan OR gaza* OR "west bank" OR philippine* OR philipine* OR phillipine* OR phillippine* OR filipino* OR filipina OR rwanda* OR rwandese OR ruanda* OR ruandese OR samoa OR polynesia* OR "sao tome and principe*" OR senegal* OR "sierra leone*" OR "solomon island" OR somali* OR "sri lanka" OR sudan* OR Syria OR tajik* OR tanzania* OR tanganyika* OR "timor leste*" OR "east timor*" OR timorese* OR togo OR togoles* OR togo's OR tonga* OR tunisia* OR uganda* OR ukrain* OR uzbek* OR "vanuatu *" OR vietnam* OR "viet nam*" OR yemen* OR zambia* OR zimbabwe* OR "southeastern asia*" OR "south eastern asia*" OR "southeast asia*" OR "south east asia*" OR "developing countr*" OR "developing nation*" OR "developing population*" OR "developing world" OR "less developed countr*" OR "less developed nation*" OR "less developed world" OR "lesser developed countr*" OR "lesser developed nation*" OR "lesser developed world" OR "under developed countr*" OR "under developed nation*" OR "under developed world" OR "underdeveloped countr*" OR "underdeveloped nation*" OR "underdeveloped world" OR "low income countr*" OR "low income nation*" OR "low income population*" OR "lower income countr*" OR "lower income nation*" OR "lower income population*" OR "underserved countr*" OR "underserved nation*" OR "underserved population*" OR "under served population*" OR "under served nation*" OR "under served population*" OR "deprived countr*" OR "deprived population*" OR "high burden countr*" OR "high burden nation*" OR "countdown countr*" OR "countdown nation*" OR "poor countr*" OR "poor nation*" OR "poor population*" OR "poor world" OR "poorer countr*" OR "poorer nation*" OR "poorer population*" OR "poorer world" OR "developing econom*" OR "less developed econom*" OR "underdeveloped econom*" OR "under developed econom*") |  |
|  |  |  |
| **EconLit (EbscoHost)** | ((TI "maternal health" OR AB "maternal health" OR SU "maternal health") OR (TI "neonatal health" OR AB "neonatal health" OR SU "neonatal health") OR (TI "infant health" OR AB "infant health" OR SU "infant health") OR (TI "newborn health" OR AB "newborn health" OR SU "newborn health") OR (TI "maternal mortality" OR AB "maternal mortality" OR SU "maternal mortality") OR (TI "infant mortality" OR AB "infant mortality" OR SU "infant mortality") OR (TI "newborn mortality" OR AB "newborn mortality" OR SU "newborn mortality") OR (TI "neonatal mortality" OR AB "neonatal mortality" OR SU "neonatal mortality") OR (TI "maternal death*" OR AB "maternal death*" OR SU "maternal death*") OR (TI "newborn death*" OR AB "newborn death*" OR SU "newborn death*") OR (TI "neonatal death*" OR AB "neonatal death*" OR SU "neonatal death*") OR (TI "infant death*" OR AB "infant death*" OR SU "infant death*") OR (TI "antenatal care" OR AB "antenatal care" OR SU "antenatal care") OR (TI "prenatal care" OR AB "prenatal care" OR SU "prenatal care") OR (TI "perinatal care" OR AB "perinatal care" OR SU "perinatal care") OR ((TI safe* OR AB safe* OR SU safe*) N2 (TI motherhood OR AB motherhood OR SU motherhood)) OR (TI "maternity care" OR AB "maternity care" OR SU "maternity care") OR ((TI "perinatal death*" OR AB "perinatal death*" OR SU "perinatal death*") OR (TI "perinatal mortality" OR AB "perinatal mortality" OR SU "perinatal mortality") OR (TI stillbirth* OR AB stillbirth* OR SU stillbirth*) OR (TI "still birth*" OR AB "still birth*" OR SU "still birth*") OR (TI stillborn OR AB stillborn OR SU stillborn) OR (TI "still born" OR AB "still born" OR SU "still born") OR (TI "F#etal death" OR AB "F#etal death" OR SU "F#etal death") OR (TI "F#etal demise" OR AB "F#etal demise" OR SU "F#etal demise")) |  |
|  | AND |  |
|  | (TI (evaluation OR monitor* OR index OR assess* OR doctrine* OR health* OR review OR framework OR strateg* OR program* OR intervention* OR development OR audit OR "confidential inquiry" OR surveillance OR localization OR scale-up)) OR (AB (evaluation OR monitor* OR index OR assess* OR doctrine* OR health* OR review OR framework OR strateg* OR program* OR intervention* OR development OR audit OR "confidential inquiry" OR surveillance OR localization OR scale-up)) OR (SU (evaluation OR monitor* OR index OR assess* OR doctrine* OR health* OR review OR framework OR strateg* OR program* OR intervention* OR development OR audit OR "confidential inquiry" OR surveillance OR localization OR scale-up)) |  |
|  | AND |  |
|  | (TI (Costs OR cost OR economic*) OR (AB (Costs OR cost OR economic*)) OR (SU (Costs OR cost OR economic*)) |  |
|  | AND |  |
|  | ((TI afghan* OR AB afghan* OR SU afghan*) OR (TI africa* OR AB africa* OR SU africa*) OR (TI algeria* OR AB algeria* OR SU algeria*) OR (TI angola* OR AB angola* OR SU angola*) OR (TI bangladesh* OR AB bangladesh* OR SU bangladesh*) OR (TI benin* OR AB benin* OR SU benin*) OR (TI bhutan* OR AB bhutan* OR SU bhutan*) OR (TI bolivia* OR AB bolivia* OR SU bolivia*) OR (TI burkina* OR AB burkina* OR SU burkina*) OR (TI burkinese* OR AB burkinese* OR SU burkinese*) OR (TI burundi* OR AB burundi* OR SU burundi*) OR (TI urundi* OR AB urundi* OR SU urundi*) OR (TI "cabo verde*" OR AB "cabo verde*" OR SU "cabo verde*") OR (TI "cape verde*" OR AB "cape verde*" OR SU "cape verde*") OR (TI cambodia* OR AB cambodia* OR SU cambodia*) OR (TI cameroon* OR AB cameroon* OR SU cameroon*) OR (TI cameroun* OR AB cameroun* OR SU cameroun*) OR (TI chad* OR AB chad* OR SU chad*) OR (TI comoro* OR AB comoro* OR SU comoro*) OR (TI comore* OR AB comore* OR SU comore*) OR (TI comorian* OR AB comorian* OR SU comorian*) OR (TI congo* OR AB congo* OR SU congo*) OR (TI "cote d'ivoir*" OR AB "cote d'ivoir*" OR SU "cote d'ivoir*") OR (TI "cote d' ivoir*" OR AB "cote d' ivoir*" OR SU "cote d' ivoir*") OR (TI "cote divoir*" OR AB "cote divoir*" OR SU "cote divoir*") OR (TI "cote d ivoir*" OR AB "cote d ivoir*" OR SU "cote d ivoir*") OR (TI "ivory coast*" OR AB "ivory coast*" OR SU "ivory coast*") OR (TI ivorian* OR AB ivorian* OR SU ivorian*) OR (TI Djibouti OR AB Djibouti OR SU Djibouti) OR (TI Egypt* OR AB Egypt* OR SU Egypt*) OR (TI "el Salvador" OR AB "el Salvador" OR SU "el Salvador") OR (TI guinea* OR AB guinea* OR SU guinea*) OR (TI equatoguinea* OR AB equatoguinea* OR SU equatoguinea*) OR (TI Eritrea OR AB Eritrea OR SU Eritrea) OR (TI eswatini* OR AB eswatini* OR SU eswatini*) OR (TI swaziland* OR AB swaziland* OR SU swaziland*) OR (TI swazi* OR AB swazi* OR SU swazi*) OR (TI swati* OR AB swati* OR SU swati*) OR (TI ethiopia* OR AB ethiopia* OR SU ethiopia*) OR (TI gambia* OR AB gambia* OR SU gambia*) OR (TI ghana OR AB ghana OR SU ghana) OR (TI haiti* OR AB haiti* OR SU haiti*) OR (TI hondura* OR AB hondura* OR SU hondura*) OR (TI india* OR AB india* OR SU india*) OR (TI indonesia* OR AB indonesia* OR SU indonesia*) OR (TI iran* OR AB iran* OR SU iran*) OR (TI kenya* OR AB kenya* OR SU kenya*) OR (TI korea* OR AB korea* OR SU korea*) OR (TI laos OR AB laos OR SU laos) OR (TI lao OR AB lao OR SU lao) OR (TI laotian* OR AB laotian* OR SU laotian*) OR (TI lebanon* OR AB lebanon* OR SU lebanon*) OR (TI Lebanese OR AB Lebanese OR SU Lebanese) OR (TI Liberia OR AB Liberia OR SU Liberia) OR (TI madagasca OR AB madagasca OR SU madagasca) OR (TI malawi* OR AB malawi* OR SU malawi*) OR (TI mali OR AB mali OR SU mali) OR (TI Micronesia* OR AB Micronesia* OR SU Micronesia*) OR (TI mauritania* OR AB mauritania* OR SU mauritania*) OR (TI mauritan* OR AB mauritan* OR SU mauritan*) OR (TI Mauritius OR AB Mauritius OR SU Mauritius) OR (TI mongol* OR AB mongol* OR SU mongol*) OR (TI morocco* OR AB morocco* OR SU morocco*) OR (TI Moroccan* OR AB Moroccan* OR SU Moroccan*) OR (TI mozambique* OR AB mozambique* OR SU mozambique*) OR (TI mozambican* OR AB mozambican* OR SU mozambican*) OR (TI Myanmar OR AB Myanmar OR SU Myanmar) OR (TI Nepal OR AB Nepal OR SU Nepal) OR (TI nicaragua* OR AB nicaragua* OR SU nicaragua*) OR (TI niger* OR AB niger* OR SU niger*) OR (TI Pakistan OR AB Pakistan OR SU Pakistan) OR (TI gaza* OR AB gaza* OR SU gaza*) OR (TI "west bank" OR AB "west bank" OR SU "west bank") OR (TI philippine* OR AB philippine* OR SU philippine*) OR (TI philipine* OR AB philipine* OR SU philipine*) OR (TI phillipine* OR AB phillipine* OR SU phillipine*) OR (TI phillippine* OR AB phillippine* OR SU phillippine*) OR (TI filipino* OR AB filipino* OR SU filipino*) OR (TI filipina OR AB filipina OR SU filipina) OR (TI rwanda* OR AB rwanda* OR SU rwanda*) OR (TI rwandese OR AB rwandese OR SU rwandese) OR (TI ruanda* OR AB ruanda* OR SU ruanda*) OR (TI ruandese OR AB ruandese OR SU ruandese) OR (TI samoa OR AB samoa OR SU samoa) OR (TI polynesia* OR AB polynesia* OR SU polynesia*) OR (TI "sao tome and principe*" OR AB "sao tome and principe*" OR SU "sao tome and principe*") OR (TI senegal* OR AB senegal* OR SU senegal*) OR (TI "sierra leone*" OR AB "sierra leone*" OR SU "sierra leone*") OR (TI "solomon island" OR AB "solomon island" OR SU "solomon island") OR (TI somali* OR AB somali* OR SU somali*) OR (TI "sri lanka" OR AB "sri lanka" OR SU "sri lanka") OR (TI sudan* OR AB sudan* OR SU sudan*) OR (TI Syria OR AB Syria OR SU Syria) OR (TI tajik* OR AB tajik* OR SU tajik*) OR (TI tanzania* OR AB tanzania* OR SU tanzania*) OR (TI tanganyika* OR AB tanganyika* OR SU tanganyika*) OR (TI "timor leste*" OR AB "timor leste*" OR SU "timor leste*") OR (TI "east timor*" OR AB "east timor*" OR SU "east timor*") OR (TI timorese* OR AB timorese* OR SU timorese*) OR (TI togo OR AB togo OR SU togo) OR (TI togoles* OR AB togoles* OR SU togoles*) OR (TI togo's OR AB togo's OR SU togo's) OR (TI tonga* OR AB tonga* OR SU tonga*) OR (TI tunisia* OR AB tunisia* OR SU tunisia*) OR (TI uganda* OR AB uganda* OR SU uganda*) OR (TI ukrain* OR AB ukrain* OR SU ukrain*) OR (TI uzbek* OR AB uzbek* OR SU uzbek*) OR (TI "vanuatu *" OR AB "vanuatu *" OR SU "vanuatu *") OR (TI vietnam* OR AB vietnam* OR SU vietnam*) OR (TI "viet nam*" OR AB "viet nam*" OR SU "viet nam*") OR (TI yemen* OR AB yemen* OR SU yemen*) OR (TI zambia* OR AB zambia* OR SU zambia*) OR (TI zimbabwe* OR AB zimbabwe* OR SU zimbabwe*) OR (TI "southeastern asia*" OR AB "southeastern asia*" OR SU "southeastern asia*") OR (TI "south eastern asia*" OR AB "south eastern asia*" OR SU "south eastern asia*") OR (TI "southeast asia*" OR AB "southeast asia*" OR SU "southeast asia*") OR (TI "south east asia*" OR AB "south east asia*" OR SU "south east asia*") OR (TI "developing countr*" OR AB "developing countr*" OR SU "developing countr*") OR (TI "developing nation*" OR AB "developing nation*" OR SU "developing nation*") OR (TI "developing population*" OR AB "developing population*" OR SU "developing population*") OR (TI "developing world" OR AB "developing world" OR SU "developing world") OR (TI "less developed countr*" OR AB "less developed countr*" OR SU "less developed countr*") OR (TI "less developed nation*" OR AB "less developed nation*" OR SU "less developed nation*") OR (TI "less developed world" OR AB "less developed world" OR SU "less developed world") OR (TI "lesser developed countr*" OR AB "lesser developed countr*" OR SU "lesser developed countr*") OR (TI "lesser developed nation*" OR AB "lesser developed nation*" OR SU "lesser developed nation*") OR (TI "lesser developed world" OR AB "lesser developed world" OR SU "lesser developed world") OR (TI "under developed countr*" OR AB "under developed countr*" OR SU "under developed countr*") OR (TI "under developed nation*" OR AB "under developed nation*" OR SU "under developed nation*") OR (TI "under developed world" OR AB "under developed world" OR SU "under developed world") OR (TI "underdeveloped countr*" OR AB "underdeveloped countr*" OR SU "underdeveloped countr*") OR (TI "underdeveloped nation*" OR AB "underdeveloped nation*" OR SU "underdeveloped nation*") OR (TI "underdeveloped world" OR AB "underdeveloped world" OR SU "underdeveloped world") OR (TI "low income countr*" OR AB "low income countr*" OR SU "low income countr*") OR (TI "low income nation*" OR AB "low income nation*" OR SU "low income nation*") OR (TI "low income population*" OR AB "low income population*" OR SU "low income population*") OR (TI "lower income countr*" OR AB "lower income countr*" OR SU "lower income countr*") OR (TI "lower income nation*" OR AB "lower income nation*" OR SU "lower income nation*") OR (TI "lower income population*" OR AB "lower income population*" OR SU "lower income population*") OR (TI "underserved countr*" OR AB "underserved countr*" OR SU "underserved countr*") OR (TI "underserved nation*" OR AB "underserved nation*" OR SU "underserved nation*") OR (TI "underserved population*" OR AB "underserved population*" OR SU "underserved population*") OR (TI "under served population*" OR AB "under served population*" OR SU "under served population*") OR (TI "under served nation*" OR AB "under served nation*" OR SU "under served nation*") OR (TI "under served population*" OR AB "under served population*" OR SU "under served population*") OR (TI "deprived countr*" OR AB "deprived countr*" OR SU "deprived countr*") OR (TI "deprived population*" OR AB "deprived population*" OR SU "deprived population*") OR (TI "high burden countr*" OR AB "high burden countr*" OR SU "high burden countr*") OR (TI "high burden nation*" OR AB "high burden nation*" OR SU "high burden nation*") OR (TI "countdown countr*" OR AB "countdown countr*" OR SU "countdown countr*") OR (TI "countdown nation*" OR AB "countdown nation*" OR SU "countdown nation*") OR (TI "poor countr*" OR AB "poor countr*" OR SU "poor countr*") OR (TI "poor nation*" OR AB "poor nation*" OR SU "poor nation*") OR (TI "poor population*" OR AB "poor population*" OR SU "poor population*") OR (TI "poor world" OR AB "poor world" OR SU "poor world") OR (TI "poorer countr*" OR AB "poorer countr*" OR SU "poorer countr*") OR (TI "poorer nation*" OR AB "poorer nation*" OR SU "poorer nation*") OR (TI "poorer population*" OR AB "poorer population*" OR SU "poorer population*") OR (TI "poorer world" OR AB "poorer world" OR SU "poorer world") OR (TI "developing econom*" OR AB "developing econom*" OR SU "developing econom*") OR (TI "less developed econom*" OR AB "less developed econom*" OR SU "less developed econom*") OR (TI "underdeveloped econom*" OR AB "underdeveloped econom*" OR SU "underdeveloped econom*") OR (TI "under developed econom*" OR AB "under developed econom*" OR SU "under developed econom*")) |  |
|  |  |  |
|  |  |  |
|  |  |  |
|  | 2012 – 2023 ; |  |
| **Sociological (Abstracts)** | TI,AB("maternal health" OR "neonatal health" OR "infant health" OR "newborn health" OR "maternal mortality" OR "infant mortality" OR "newborn mortality" OR "neonatal mortality" OR "maternal death*" OR "newborn death*" OR "neonatal death*" OR "infant death*" OR "antenatal care" OR "prenatal care" OR "perinatal care" OR (safe* NEAR/2 motherhood) OR "maternity care" OR "perinatal death*" OR "perinatal mortality" OR stillbirth* OR "still birth*" OR stillborn OR "still born" OR "F*etal death" OR "F*etal demise") |  |
|  | AND |  |
|  | (TI,AB(evaluation) OR TI,AB(monitor*) OR TI,AB(assess*) OR TI,AB(doctrine*) OR TI,AB(review) OR TI,AB(framework) OR TI,AB(strateg*) OR TI,AB(intervention*) OR TI,AB(development) OR TI,AB(audit) OR TI,AB("confidential inquiry") OR TI,AB(surveillance) OR TI,AB(localization) OR TI,AB(scale-up)) |  |
|  | AND |  |
|  | TI,AB(Costs) OR TI,AB(cost) OR TI,AB(economic*) |  |
|  | AND |  |
|  | TI,AB(afghan* OR africa* OR algeria* OR angola* OR bangladesh* OR benin* OR bhutan* OR bolivia* OR burkina* OR burkinese* OR burundi* OR urundi* OR "cabo verde*" OR "cape verde*" OR cambodia* OR cameroon* OR cameroun* OR chad* OR comoro* OR comore* OR comorian* OR congo* OR "cote d'ivoir*" OR "cote d' ivoir*" OR "cote divoir*" OR "cote d ivoir*" OR "ivory coast*" OR ivorian* OR Djibouti OR Egypt* OR "el Salvador" OR guinea* OR equatoguinea* OR Eritrea OR eswatini* OR swaziland* OR swazi* OR swati* OR ethiopia* OR gambia* OR ghana OR haiti* OR hondura* OR india* OR indonesia* OR iran* OR kenya* OR korea* OR laos OR lao OR laotian* OR lebanon* OR Lebanese OR Liberia OR madagasca OR malawi* OR mali OR Micronesia* OR mauritania* OR mauritan* OR Mauritius OR mongol* OR morocco* OR Moroccan* OR mozambique* OR mozambican* OR Myanmar OR Nepal OR nicaragua* OR niger* OR Pakistan OR gaza* OR "west bank" OR philippine* OR philipine* OR phillipine* OR phillippine* OR filipino* OR filipina OR rwanda* OR rwandese OR ruanda* OR ruandese OR samoa OR polynesia* OR "sao tome and principe*" OR senegal* OR "sierra leone*" OR "solomon island" OR somali* OR "sri lanka" OR sudan* OR Syria OR tajik* OR tanzania* OR tanganyika* OR "timor leste*" OR "east timor*" OR timorese* OR togo OR togoles* OR togo's OR tonga* OR tunisia* OR uganda* OR ukrain* OR uzbek* OR "vanuatu *" OR vietnam* OR "viet nam*" OR yemen* OR zambia* OR zimbabwe* OR "southeastern asia*" OR "south eastern asia*" OR "southeast asia*" OR "south east asia*" OR "developing countr*" OR "developing nation*" OR "developing population*" OR "developing world" OR "less developed countr*" OR "less developed nation*" OR "less developed world" OR "lesser developed countr*" OR "lesser developed nation*" OR "lesser developed world" OR "under developed countr*" OR "under developed nation*" OR "under developed world" OR "underdeveloped countr*" OR "underdeveloped nation*" OR "underdeveloped world" OR "low income countr*" OR "low income nation*" OR "low income population*" OR "lower income countr*" OR "lower income nation*" OR "lower income population*" OR "underserved countr*" OR "underserved nation*" OR "underserved population*" OR "under served population*" OR "under served nation*" OR "under served population*" OR "deprived countr*" OR "deprived population*" OR "high burden countr*" OR "high burden nation*" OR "countdown countr*" OR "countdown nation*" OR "poor countr*" OR "poor nation*" OR "poor population*" OR "poor world" OR "poorer countr*" OR "poorer nation*" OR "poorer population*" OR "poorer world" OR "developing econom*" OR "less developed econom*" OR "underdeveloped econom*" OR "under developed econom*") |  |
| **Global Index Medicus** | (tw:(("maternal health" OR "neonatal health" OR "infant health" OR "newborn health" OR "maternal mortality" OR "infant mortality" OR "newborn mortality" OR "neonatal mortality" OR "maternal death*" OR "newborn death*" OR "neonatal death*" OR "infant death*" OR "antenatal care" OR "prenatal care" OR "perinatal care" OR (safe* NEAR/2 motherhood) OR "maternity care" OR "perinatal death*" OR "perinatal mortality" OR stillbirth* OR "still birth*" OR stillborn OR "still born" OR "Fetal death" OR "Fetal demise" OR "Foetal death" OR "Foetal demise"))) AND (tw:(evaluation OR monitor* OR index OR assess* OR doctrine* OR health* OR review OR framework OR strateg* OR program* OR intervention* OR development OR audit OR "confidential inquiry" OR surveillance OR localization OR scale-up))) AND (tw:(Costs OR cost OR economic*)) |  |
| Note: Search strategy created in collaboration with Centers for Disease Control and Prevention library services. Search was conducted by library services. | |  |
