# Supplementary material for: Cost of implementation and maintenance of maternal and perinatal death surveillance and response: a scoping review
Source: BMC Pregnancy Childbirth. 2025 Oct 6;25(Suppl 1):1016. doi: 10.1186/s12884-025-08181-z (PMC12498452; doi:10.1186/s12884-025-08181-z)
Supplement: Supplementary file 2 — Supplementary Material 2 [file 12884_2025_8181_MOESM2_ESM.docx]

| **Supplementary Table 2. Total and itemized costs in the currency originally reported for articles that described cost categories, *n* = 3** | | | | | | | | | |
| --- | --- | --- | --- | --- | --- | --- | --- | --- | --- |
| **Author** | **Joshi et al.^a^ [26]** | | | **Biswas et al.^b^ [27]** | | | **Serbanescu et al.^c^ [29]** | | |
| **Cost category** | **Year 1 costs (2003, INR)** | **Year 2 costs (2004–2007, INR)** | **Year 3 costs (2004–2007, INR)** | **Year 1 costs (2010, BDT)** | **Year 2 costs (2011, BDT)** | **Year 3 costs (2012, BDT)** | **Year 1 costs (2013, UGX)** | **Year 2 costs (2014, UGX)** | **Year 3 costs (2015, UGX)** |
|  |  |  |  |  |  |  |  |  |  |
| Training | 67,025.00 | 31,012.50 | 31,012.50 | 75,118.00 | 75,118.00 | 75,118.00 | 371,908,800.00 | Not applicable | 36,000,000.00 |
| Tool development | Included in training | Included in training | Included in training | 214,589.00 | 214,589.00 | 214,589.00 | Included in project management | Included in project management | Included in project management |
| Meetings | Not measured | Not measured | Not measured | 241,136.00 | 257,852.00 | 202,515.00 | 57,659,520.00 | 57,659,520.00 | 57,659,520.00 |
| Infrastructure/capacity | 101,342.60 | 16,942.60 | 16,942.60 | 2,649,045.00 | 2,012,791.00 | 1,396,470.00 | 603,040,804.00 | Included in data collection | Included in data collection |
| Project management | 341,742.00 | 338,862.00 | 338,862.00 | 6,485,145.00 | 4,382,658.00 | 4,175,852.00 | 1,910,000.00 | 25,365,333.00 | 25,365,333.00 |
| Community data collection | 623,400.00 | 435,900.00 | 435,900.00 | 553,000.00 | 728,700.00 | 260,400.00 | 160,978,080.00 | 504,276,480.00 | 504,276,480.00 |
| Facility data collection | Not measured | Not measured | Not measured | 52,600.00 | 103,550.00 | 319,950.00 | Not measured | Not measured | Not measured |
| Monitoring | Not measured | Not measured | Not measured | 1,348,106.00 | 1,412,531.00 | 723,207.00 | 7,250,000.00 | 12,517,500.00 | 12,517,500.00 |
| **Total cost (per year)** | **1,133,509.60** | **822,717.10** | **822,717.10** | **11,618,739.00** | **9,187,789.00** | **7,368,101.00** | **1,202,747,204.00** | **599,818,833.00** | **635,818,833.00** |
| Abbreviations: INR, Indian Rupee; BDT, Bangladesh Taka; UGX, Ugandan Shilling. | | | | | | | | | |
| a. Joshi et al. total reported costs by category were taken from the published paper. The prospective mortality surveillance of deaths (child and adult deaths) described by the authors ran for 4 years. Costs were reported in INR at the 2003 currency rate for all years. Costs were reported for the start-up year (2003/2004) and the following 3 years. Maintenance costs for 3 years were not reported annually but for the whole 3-year period. We averaged these costs to obtain annualized maintenance costs by dividing the published category costs for maintenance by 3. Summation errors in the Joshi et al. reported tables were noted and corrected. | | | | | | | | | |
| b. Biswas et al. total reported costs by category were provided by author in BDT at the 2012 currency rate for all 3 years. Itemized costs that made up cost categories were further clarified with the author. | | | | | | | | | |
| c. Serbanescu et al. total reported costs by category were provided by author in UGX at the 2013 currency rate for all 3 years. Facility data collection costs were not measured. Itemized costs that made up cost categories were further clarified with the author. | | | | | | | | | |
