# Supplementary material for: Cost of implementation and maintenance of maternal and perinatal death surveillance and response: a scoping review
Source: BMC Pregnancy Childbirth. 2025 Oct 6;25(Suppl 1):1016. doi: 10.1186/s12884-025-08181-z (PMC12498452; doi:10.1186/s12884-025-08181-z)
Supplement: Supplementary file 4 — Supplementary Material 4 [file 12884_2025_8181_MOESM4_ESM.docx]

| **Supplementary Table 3b. Year 2 total and itemized costs overall, per death reviewed, and per capita in USD 2024 for articles that described cost categories, *n* = 3** | | | | | | | | | |
| --- | --- | --- | --- | --- | --- | --- | --- | --- | --- |
|  | **Year 2 overall cost** | | | **Year 2 cost per death reviewed** | | | **Year 2 cost per capita** | | |
| Author | Joshi et al.^a^ [26] | Biswas et al.^b^ [27] | Serbanescu et al.^c^ [29] | Joshi et al | Biswas et al | Serbanescu et al | Joshi et al | Biswas et al | Serbanescu et al |
| Population and deaths reviewed | 185,628 | 1,400,000 | 1,278,004 | 1,430 | 1,667 | 177 | 185,628 | 1,400,000 | 1,278,004 |
| **Cost category** |  |  |  |  |  |  |  |  |  |
| Training | $ ,611.41 | $3,652.76 | Not applicable | $ 3.22 | $2.19 | Not applicable | $ 0.02 | $0.00 | Not applicable |
| Tool maintenance | Included in training | $10,434.82 | Included in project management | Included in training | $6.26 | Included in project management | Included in training | $0.01 | Included in project management |
| Meetings | Not measured | $5,409.65 | $ 68,175.90 | Not measured | $3.25 | $ 385.17 | Not measured | $0.00 | $ 0.05 |
| Infrastructure and capacity building | $ 2,519.29 | $97,875.96 | Included in data collection | $ 1.76 | $58.71 | Included in data collection | $ 0.01 | $0.07 | Included in data collection |
| Project management | $50,387.21 | $213,115.45 | $ 29,991.65 | $ 35.24 | $127.84 | $ 169.44 | $ 0.27 | $0.15 | $ 0.02 |
| Community data collection | 64816.31 | $38,998.94 | $ 596,250.20 | $ 45.33 | $23.39 | $ 3,368.65 | $ 0.35 | $0.03 | $ 0.47 |
| Facility data collection | Not applicable | $8,599.78 | Not applicable | Not applicable | $5.16 | Not applicable | Not applicable | $0.01 | Not applicable |
| Monitoring | Not measured | $68,687.13 | $ 14,800.54 | Not measured | $41.20 | $ 83.62 | Not measured | $0.05 | $ 0.01 |
| Total cost per unit |  |  |  | $ 85.55 | $268.01 | $ 4,006.88 | $ 0.66 | $0.32 | $ 0.55 |
| Total cost per year | $ 122,334.22 | $446,774.49 | $ 709,218.29 |  |  |  |  |  |  |
| Abbreviations: USD, United States Dollar; INR, Indian Rupee; BDT, Bangladesh Taka; UGX, Ugandan Shilling. | | | | | | | | | |
| Notes: All costs are converted from the original currency and year to $USD 2024 and adjusted for purchasing power parity and gross domestic product using the CCEMG-EPPI-Centre Cost Converter v.1.4 (ioe.ac.uk) tool. "Not applicable" indicates that the surveillance did not incur a cost category. | | | | | | | | | |
| a. Joshi et al. total reported costs by category were taken from the published paper. The prospective mortality surveillance of deaths (child and adult deaths) described by the authors ran for 4 years. Costs were reported in INR at the 2003 currency rate for all years. Costs were reported for the start-up year (2003/2004) and the following 3 years. Maintenance costs for 3 years were not reported annually but for the whole 3-year period. We averaged these costs to obtain annualized maintenance costs by dividing the published category costs for maintenance by 3. Summation errors in the Joshi et al. reported tables were noted and corrected. Joshi et al. report the total number of deaths reviewed over a 4-year period (child and adult deaths); number of deaths reviewed per year were calculated by authors using the published annual costs per death reviewed. We then computed annual costs per death reviewed and per capita using the population provided in the article. | | | | | | | | | |
| b. Biswas et al. total reported costs by category were provided by author in BDT at the 2012 currency rate for all 3 years. Itemized costs that made up cost categories were further clarified with the author. Biswas et al. reviewed community and facility maternal and neonatal deaths and still births. Total number of deaths per death type and year were not reported in the paper but obtained from the author. For all 3 years, we then calculated the annual costs per death reviewed using the reported total costs and the annual number of deaths reviewed; we also calculated the annual costs per capita using the population provided in the article. | | | | | | | | | |
| c. Serbanescu et al. total reported costs by category were provided by author in UGX at the 2013 currency rate for all 3 years. Facility data collection costs were not measured. Itemized costs that made up cost categories were further clarified with the author. Serbanescu et al. reviewed only maternal deaths in the community. Serbanescu et al. did not report total number of deaths reviewed; this was obtained from authors. For all 3 years, we then calculated the annual costs per death reviewed using the reported total costs and the annual number of deaths reviewed; we also calculated the annual costs per capita using the population provided in the article. | | | | | | | | | |
